# Association of attitudes towards genetically modified food among young adults and their referent persons

**DOI:** 10.1371/journal.pone.0211879

**Published:** 2019-02-04

**Authors:** Stephan Brosig, Miroslava Bavorova

**Affiliations:** 1 Leibniz-Institute of Agricultural Development in Transition Economies, Halle (Saale), Germany; 2 Department of Economic Development, Faculty of Tropical AgriSciences, Czech University of Life Sciences, Prague, Czech Republic; University of Naples Federico II, ITALY

## Abstract

Most research on consumer attitudes does not consider that attitudes are likely influenced by people with whom we have some relationship even though socioeconomic, psychological and political theories recognize the importance of referent individuals’ opinions in attitude formation. Knowledge on the role of referent individuals’ opinions in attitude formation could improve the understanding of consumer acceptance of foods frequently associated with health or other concerns. This article examines the association of attitudes towards genetically modified (GM) crops and foods between young adults and their referent individuals using data collected in 2016 via surveys from the Czech Republic, Russia and Ukraine. Loglinear models of cell counts in contingency tables reveal a positive association of GM food attitudes between young adults and their referent individuals. This association was stronger in Russia and the Czech Republic than it was in Ukraine and stronger between female young adults and their referent individuals than between males and their referent individuals. Concordance in GM food attitudes with mothers is significantly stronger than concordance with best friends but not significantly different from concordance with fathers.

## Introduction

Genetically modified (GM) crops and foods, i.e., the usage of genetically modified organisms (GMOs) in agriculture and food production, have been widely debated during all generations of GM technology, and consumers’ attitudes towards GMOs are the focus of multiple studies [1–6]. This paper focuses on food consumers; we investigate the formation of consumer attitudes towards GM crops and foods, specifically the association of attitudes between young adults and their nearest referent persons.

The relevance of referent persons in consumer attitudes, consumer preferences and consumer decisions has been studied in different contexts and across disciplines. Concepts and findings from relationship science are related to consumer behavior in relationships in a series of contributions from consumer psychology compiled by Priester and Petty [7]. Aside from an extensive review of the relevant literature the authors provide a framework for the analysis of dyadic consumer data, highlight a range of theories explaining consumer behavior in relationships and identify directions for advancing our understanding of consumer attitudes and consumer decisions in relationship contexts [8–12].

A number of consumer studies show that parents and peers are two key sources likely to impact individuals’ attitudes towards foods [e.g. 13, 14]. Further, political science studies confirm the transmission of political beliefs from parents to their children, particularly if the family is highly politicized and parents provide consistent cues over time [15]. Antonopoulou, Papadas, and Targoutzidis [16] show that political perceptions are a significantly influential factor regarding consumers’ attitudes towards GM food. While consumer and political science studies have mainly analyzed the effects that parents have on the attitudes of children under 18, we explore with regard to an older age group, young adults, how their attitudes towards new technologies in food production are associated with the (perceived) attitudes of their parents and friends.

One of the most popular sociopsychological models for understanding and predicting human behavior, the theory of planned behavior (TPB) by Ajzen [17, 18], considers the importance of individuals' social relations. Altogether, the TPB identifies three determinants of intentions required to perform a certain behavior: the person's own valuation of the behavior ("*attitude"*), his or her assessment of the valuation of the behavior by those of importance to him or her ("*subjective norms"*) and his or her assessment of the ease or difficulty of performing the behavior ("*behavioral control"*). Subjective norms, the determinant of particular interest in this study, relate to perceived social influences/pressures regarding a certain behavior [18]. “Those of importance” have been referred to as “referent individuals” [18] and “significant others”.

The TPB has emerged as a frequently used tool for analyzing consumer behavior, and accordingly, there exists a rich literature, particularly with a focus on food consumption and health behavior. For example, Ajzen [19] discusses the application of the TPB to food consumption decisions. Multiple works have applied the theory and various extended versions in marketing and behavioral studies to explain consumer behavior towards food [cf. e.g. 20–25]. Tarkiainen and Sundqvist [25] emphasize that strong effects of subjective norms on attitudes have been identified with regard to behavior that involves some kind of ethical decision. Such an ethical dimension is commonly associated with GM attitudes, and Sparks, Shepherd, and Frewer [23] account for this in their study on GM food attitudes by measuring the perceived ethical obligation and GM food attitudes of significant others. Prati, Pietrantoni, and Zani [22] developed a model of intention to consume GM food based on the TPB. They find two separate aspects of subjective norms that directly impact intentions to consume GM food: the degree to which referent persons (are believed to) *care about* GM food [24], and the degree to which referent persons *approve* of GM food [23]. The authors also included trust, perceived benefits and risks as additional explanatory factors and found in their sample of Italian consumers that attitude was the most important factor among the three predictors of intention suggested by the TPB. Attitude, in turn, was predicted by the perceived risks and benefits. Also Bagozzi, Wong, Abe, and Bergami [26] consider subjective norms and find their impact on the intention to visit fast-food restaurants to be strong for Chinese consumers and weaker for American, Italian and Japanese consumers. The framework used in their study is the theory of reasoned action [27, 28], a predecessor of the TPB.

Fishbein and Ajzen [28] and Chang [29] have noted the possibility of direct links between subjective norms and attitudes, and Tarkiainen and Sundqvist [25] challenge the view that attitudes and subjective norms should be regarded as independent determinants of intentions. The latter authors studied the relationships between subjective norms and attitudes and the subsequent intention of buying organic food by using a sample of 200 Finnish consumers. Employing a structural equation model (SEM) framework, Tarkiainen and Sundqvist found that subjective norms do in fact affect attitude formation (and hence affect buying intentions not only directly but also via their impact on attitudes).

Similarly, our empirical study examines links between attitudes and subjective norms. Following Sparks, Shepherd, and Frewer [23], we proxy subjective norms by the perceived attitudes of referent individuals, and we measure the association between young adults' GM food attitudes and the attitudes they perceive their referent persons to have. In this way, a potential impact of subjective norms on attitudes can be identified.

Specifically, we study the following research questions:

1. Is there an association between the GM food attitudes of young adults and the attitudes of their parents and friends?
2. Does the association of GM food attitudes between young adults and their parents and peers differ between countries (Russia, the Czech Republic and Ukraine)?
3. Does the association of GM food attitudes between young adults and their referent persons differ by the gender of the young adult and is it different towards their fathers and mothers?

Our explorative study uses loglinear models of cell counts in contingency tables to test the association regarding GM food attitudes between young adults and their referent persons. The GM approval ratings of both the young adults and their referent persons are modeled as response variables with potentially mutual impacts on each other, while country and gender are considered explanatory factors.

Our study region comprises three postsocialist countries: the Czech Republic, Russia and Ukraine. While the results of systematic research on GM attitudes and their formation in Central and Eastern European countries are rare, surveys by public opinion centers in several transition countries have provided initial insights. They address the extent of public attention on GM food, the prevailing level of factual knowledge on GM food as well as the general approval or disapproval of GM food in the population. For the Czech Republic, the results of a 2016 opinion poll conducted among 1005 respondents older than 15 years showed that over 80% were aware of GM food as an issue, but only 50% had a clear concept of the term. Most of the respondents reported not being interested in this topic and that they never or seldomly check the information about genetic modification that is provided on food labels [30]. Twenty-six percent of the respondents showed a critical attitude by indicating that they would rather not buy GM food if they could identify it.

According to an opinion poll conducted in Russia in 2014, more than 80% of the respondents supported a ban of GM products, and 82% agreed with the statement “Products with GMOs cause harm to human health” [31]. In Ukraine, the market research company Research & Branding Group [32] conducted a nationwide public opinion survey with 2176 respondents in all regions in 2012. The study found that the majority of Ukrainians (71%) believe that GMOs are a relevant topic. Almost three-quarters of respondents said they know what GMO means; 80% of those who knew what GMO meant believed that GMO products endanger human health.

The results of these opinion surveys are not fully comparable, particularly because of the different wording of the questions that were asked. However, the results in general suggest that the fear that GM food can endanger human health is broadly spread in the population of Russia and Ukraine and to a much lower extent in the Czech Republic.

The purpose of this study is to provide empirical insights into the role of referent persons in the formation of consumer attitudes towards GM foods. A better understanding of attitude associations between referent groups can help improve strategies of communicating information on GM benefits and risks. If such an association is found, it implies that addressing information to a particular group, for example, a particular generation, will also result in predictable changes in GM food approval in other groups. Information of this type can provide insights valuable to stakeholders in agrifood sectors that are economically and politically highly significant in Central and Eastern European countries. From a methodological perspective, the empirical strength of associations between subjective norms and attitudes has implications for further research, as endogeneity of attitudes needs to be accounted for in the specification of empirical models of behavioral intentions.

## Material and methods

### Data

Our analyses are based on survey data that were collected online in October and November 2016 in the Czech Republic, the Russian Federation, and Ukraine [33]. Ten students from the Czech University of Life Sciences carried out the online survey under our guidance. Convenience sampling was used to compile the sample: the students, natives of the Czech Republic (6 students), the Russian Federation (2 students) and Ukraine (2 students), approached persons from their respective home countries via online social networks (Facebook, VKontakte, and LinkedIn). These persons were sent an invitation containing basic explanations about the survey (the initiators of the survey, its purpose) and the planned utilization of the data (including information on confidentiality) as well as a link to the online survey form. Full compliance of the survey with the ethical standards of the Ethics Committee of the Leibniz Institute of Agricultural Development in Transition Economies (IAMO) was confirmed by that committee (Ethical Clearance Statement No 2018.04). As the persons approached were informed that their participation was voluntary and fully anonymous their participation provides an implicit statement of informed consent. Our analyses were restricted to adults between 18 and 30 years old who we labeled as "young adults", a total of 382 persons (217 from the Czech Republic, 113 from Russia, and 52 from Ukraine). We focused on respondents up to the age of 30 years based on our assumption that their relationships with their parents are still relatively close, closer than for adults of higher age, whose close referent persons more likely consist of their own partners and children. Given that representativeness of the sample was not a major requirement for this first exploration of GM food attitude associations between referent persons, we decided to use the convenience sampling approach described. This approach allowed us to acquire a sufficiently large number of respondents within our budgetary limits. Table 1 provides information on the distribution by gender and on the frequency, as perceived by the respondents, that GM food topics are addressed in the media and public discourse.

**Table 1:** Composition of the sample.

		Czech Republic	Russian Federation	Ukraine
Number of respondents		217	113	52
Gender				
	Male [percent]	33.6	37.2	61.5
	Female [percent]	66.4	62.8	38.5
Frequency of hearing news about GMOs				
	Rarely or very rarely [percent]	10.6	38.9	50
	Sometimes [percent]	17.5	42.5	23.1
	Often or very often [percent]	71.9	18.6	26.9

Females were moderately overrepresented in the sample of the Czech and Russian respondents and underrepresented in the sample of Ukrainian respondents. Nearly 72% of the respondents from the Czech Republic heard news about GM crops often or very often; these frequencies were only 19% in Russia and 27% in Ukraine.

The 20-item questionnaire covered sociodemographic characteristics of the respondents and 13 items related to food produced by using GMOs. From the latter group of items, we used only those on the general attitude towards GMOs in this study. The questionnaire was prepared in English and translated into Czech, Russian and Ukrainian. The survey instrument is provided in the supplementary S1 Appendix.

### Empirical approach

We examine the distribution of attitudes towards GMO crops and food among young adults (YAs) and their referent persons (RPs), that is, their fathers, mothers, and best friends. The degree of general approval of GMOs was measured according to responses to the question “What do you [does your father/mother/friend] think about GM crops in general?” The five response categories were “GM crops are very bad”, “GM crops are bad”, “Attitude towards GM crops is neutral”, “GM crops are good”, and “GM crops are very good”. These response categories were mapped to ordinally scaled categorical variables with 5 approval levels. We considered two approval variables, ***y*** and ***z***, measuring GM crop approval by the two members of a dyad, a young adult who is the respondent in the survey and the referent person considered, respectively. The structure of these dyadic data is that of a (nonreciprocal) one-with-many design with distinguishable partners: one perceiver (the young adult) and three targets (mother, father, and best friend, respectively)[34]. Information on the referent persons' GM attitudes enters the analysis as perceived by the young adults rather than as stated by the referent persons themselves. This is appropriate for analyzing the young adults' attitude formation because it is an individual's subjective *perception* of a partner's attitude rather than the partner's *true* attitude that may influence the individual's attitude [10].

**Fig 1:**
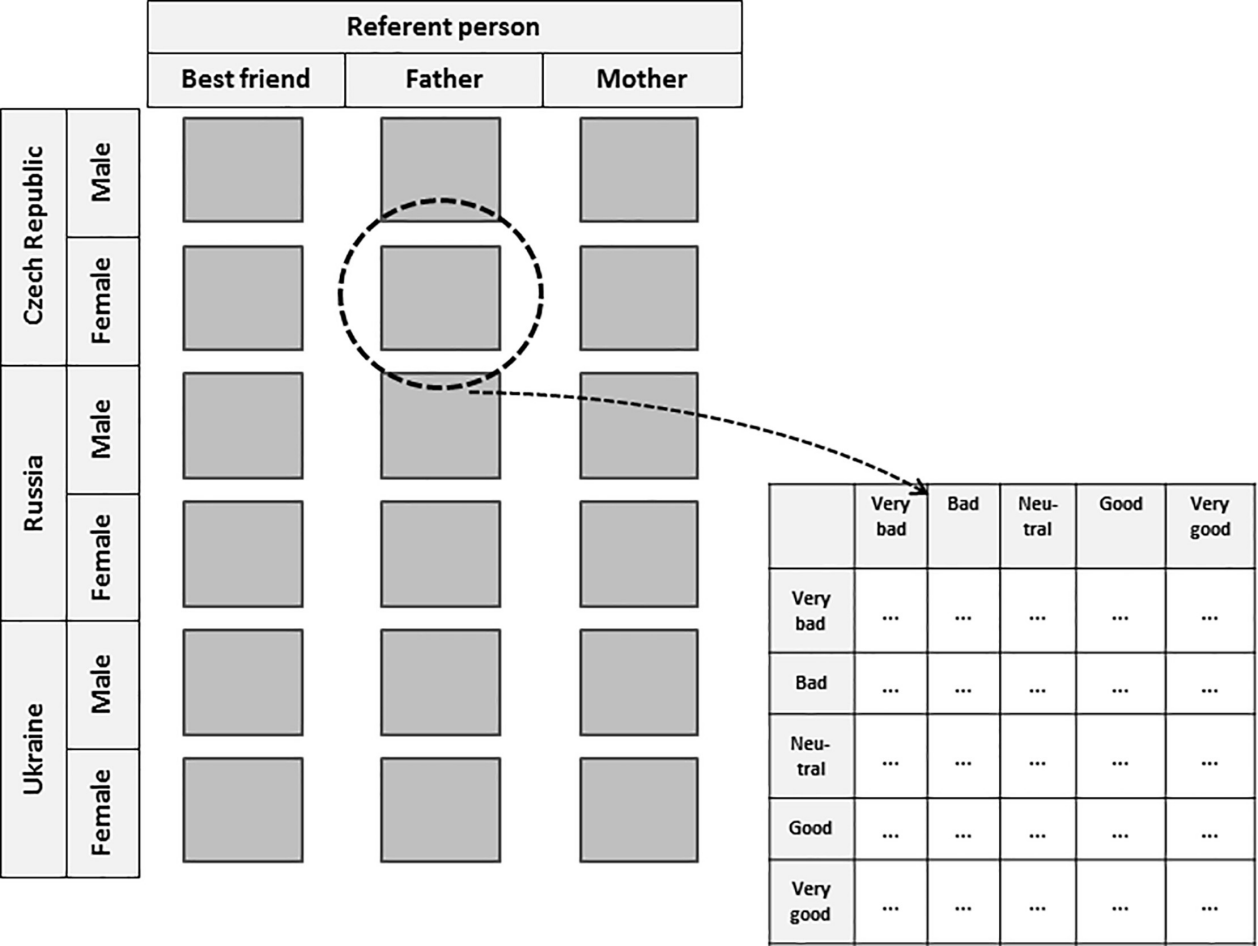
Structure of the 5-way contingency table.

The joint distribution of the two variables, ***y*** and ***z***, can be reflected in two-way contingency tables; in particular, high cell counts on or near the main diagonal of such tables correspond to positive associations between the variables. The degree of GM food approval is then likely to be similar between the YA and their RP. High counts in cells far from the main diagonal signal negative associations, meaning discordance between the YA and their RP regarding GM food approval. Heterogeneity of association patterns between the different groups of subjects can be represented in multiway contingency tables. We examined whether the association varies across the levels of three explanatory variables: (i) the type of referent person (***R***), with factor levels "father", "mother", and "best friend", (ii) the country of residence of the YA (***C***), with levels "Czech Republic", "Russian Federation", and "Ukraine" and (iii) the gender of the respondent (***G***). We hence had a five-dimensional contingency table reflecting the bivariate distribution of ***y*** and ***z*** conditional on the three nominally scaled factor variables, ***R***, ***C***, and ***G***; we aimed to assess the impact of the explanatory variables on the association of GM food attitudes between YAs and their RPs. Fig 1 illustrates the structure of this contingency table of 5*5*3*3*2 = 450 cells. (The positioning of the levels of the explanatory variables ***C*** and ***G*** in rows and ***R*** in columns is arbitrary and does not convey any meaning.) The complete contingency table is available online in the supplementary S2 Appendix.

To address our research questions, i.e., to assess the association of GMO attitudes between YAs and their RPs and to identify the impact of the factor variables, we combined a descriptive approach and a model-based analysis. We first described the univariate distributions of GMO approval by country and the distribution of the discrepancies between approval levels within dyads. This provides intuitive access to the issue of attitude association in relationships for the three countries and different groups. The analytical approach rigorously measures the association between GMO approval of YAs and RPs, making full use of the bivariate distributions of ***y*** and ***z*** in each level of the factors ***R***, ***C***, and ***G*** and without making a priori assumptions on the direction of influence within the dyads. This approach uses loglinear models of cell counts in the contingency table described above. Models of this type have been used frequently to "measure the strength of interactions (associations) among a set of categorical variables without conceptually distinguishing between response (dependent) and explanatory (independent) variables" [35]. This model type is appropriate to analyze dyadic data and, in particular, to trace associations between two or several categorical variables when a causal direction between these variables is not clear a priori [34, 36]. Examples from food consumer research include Dumitrescu, Shaw Hughner, and Shultz [37], Giudici and Passerone [38], Nedungadi [39], Velasco, Salgado-Montejo, Marmolejo-Ramos, and Spence [40], and Wan, Woods, Jacquot, Knoeferle, Kikutani, and Spence [41]. Furthermore, Gaskell, Bauer, Durant, and Allum [42] analyze survey data using loglinear models to compare GMO attitudes between Europe and the United States by focusing on associations with press coverage, trust in regulatory frameworks, and knowledge of GMOs. Loglinear models can reflect structural patterns of multivariate distributions, particularly positive or negative associations (or independence) between some variables, and these associations may be homogeneous or heterogeneous across levels of other variables. (Structural equation models (SEMs) are another option for analyzing multivariate relationships without prior assumption on causal directions, but we decided to use loglinear modeling since we focus on associations rather than formally setting up structural and measurement models. A further alternative, which is analyzing rank correlations between GM food approval levels and a statistical comparison of these correlations between pairs of groups, could also have revealed intergroup differences in the dyadic associations, but it would have entailed a loss of efficiency compared to the multivariate full-sample approach of the loglinear model.)

We estimated the parameters of a loglinear model of cell counts in the five-way contingency table mentioned above. The (logs of) cell counts *μ*_*i j k l m*_ are modeled in terms of the two response variables, ***y***_***i***_ and ***z***_***j***_, and the explanatory variables, ***R***_***k***_, ***C***_***l***_, and ***G***_***m***_. A recommendable treatment of loglinear models of the type used here is found in chapters 9 and 10 of Agresti [36]. Our specification procedure starts from a general form allowing for up to three-way heterogeneous interaction among the five involved variables. Hence, the association between any two levels of two variables is captured in a separate parameter for each level of a third variable, but this parameter is assumed to be constant across levels of the remaining two variables. In the notation used, for example, by Agresti [36], this loglinear model is

$$\begin{array}{l} \ln\mu_{ijklm}=\lambda +\lambda_{i}^{y}+\lambda_{j}^{z}+\lambda_{k}^{R}+\lambda_{l}^{C}+\lambda_{m}^{G}\\ +\lambda_{ij}^{yz}+\lambda_{i\kappa }^{yR}+\lambda_{il}^{yC}+\lambda_{im}^{yG}+\lambda_{jk}^{zR}+\lambda_{jl}^{zC}+\lambda_{jm}^{zG}+\lambda_{kl}^{RC}+\lambda_{km}^{RG}+\lambda_{lm}^{CG}\\ +\lambda_{ijk}^{yzR}+\lambda_{ijl}^{yzC}+\lambda_{ijm}^{yzG}+\lambda_{ikl}^{yRC}+\lambda_{ikm}^{yRG}+\lambda_{ilm}^{yCG}+\lambda_{jkl}^{zRC}+\lambda_{jkm}^{zRG}+\lambda_{jlm}^{zCG}+\lambda_{klm}^{RCG} \end{array}$$ (1)

with a constant and parameters for the main effects in the first line, for two-way interactions in the second line, and for three-way interactions in the third line.

This model is expensive in terms of the number of parameters to be estimated, and it does not provide readily interpretable results regarding the association of GMO attitudes between YAs and RPs, as the interaction parameters are specific to variable levels. Our specification procedure hence proceeds by restricting the model in two ways.

First, we represent the association between the two ordinally scaled GM food approval variables ***y*** and ***z*** more parsimoniously than by using the general categorical interaction effect parameters $\lambda_{ij}^{yz}$. Instead, we use a linear-by-linear (L×L) term *ß v*^***y***^ *v*^***z***^, with *v*^***y***^ and *v*^***z***^ denoting the *level scores*, that is, monotonously increasing numbers associated with the levels of ***y*** and ***z***. (We use the sequence of natural numbers, 1, 2, …, N, to score the N levels, as is done in many other applications, whereas other monotonically increasing sequences, e.g., those with varying increments, are preferred in some situations, or the scores are specified as parameters to be estimated.) Such a replacement of general categorical interaction terms $\lambda_{ij}^{yz}$ (involving (*I*-1)*(*J*-1) parameters per each but one level of explanatory variables ***R***, ***C***, and ***G***) by the L×L term (with only one parameter, *ß*_*k*_, per each but one level of each explanatory variable) means imposing a restriction that is appropriate if (a) the level scores of ***y*** and ***z*** can be meaningfully interpreted as values of cardinally-scaled variables, and (b) the relationship between the cell counts ***y*** and ***z*** can be approximated by a function that is linear in the levels of any one of the two variables at fixed levels of the other variable. In our study, neither of these preconditions could be justified on theoretical grounds. Furthermore, changes in the model fit due to imposing the L×L restriction are not insignificant according to LR tests. (Maximum likelihood estimation was performed throughout our study using the GENMOD procedure of SAS, Version 13.1.) However, the fit of our final L×L model is acceptable judging by the standardized Pearson residuals, which were below 4, and more than 90% of the residuals were below 3. (This is further discussed in the next section.) This implies an acceptable fit for an estimation fitting 450 cell counts. As a second step of model simplification, we employed a backward selection process to restrict the three-way interaction terms to zero, where LR tests suggest these restrictions to be consistent with the data.

The final model that emerged from this selection process was

$$\begin{array}{l} \ln\mu_{ijklm}=\lambda +\lambda_{i}^{y}+\lambda_{j}^{z}+\lambda_{k}^{R}+\lambda_{l}^{C}+\lambda_{m}^{G}\\ +\beta v^{y}v^{z}+\lambda_{i\kappa }^{yR}+\lambda_{il}^{yC}+\lambda_{im}^{yG}+\lambda_{jk}^{zR}+\lambda_{jl}^{zC}+\lambda_{jm}^{zG}+\lambda_{kl}^{RC}+\lambda_{km}^{RG}+\lambda_{lm}^{CG}\\ +\beta_{k}^{R}v^{y}v^{z}+\beta_{l}^{C}v^{y}v^{z}+\beta_{m}^{G}v^{y}v^{z}+\lambda_{klm}^{RCG} \end{array}$$ (2)

We estimated its parameters using ML methods, and the focus of our interest was on the *ß* parameters, which provide information on the association of GM food approval between YAs and their RPs and on how such association varies by the type of referent person, country and gender of the young adult.

## Results

### Descriptive analysis

**Fig 2:**
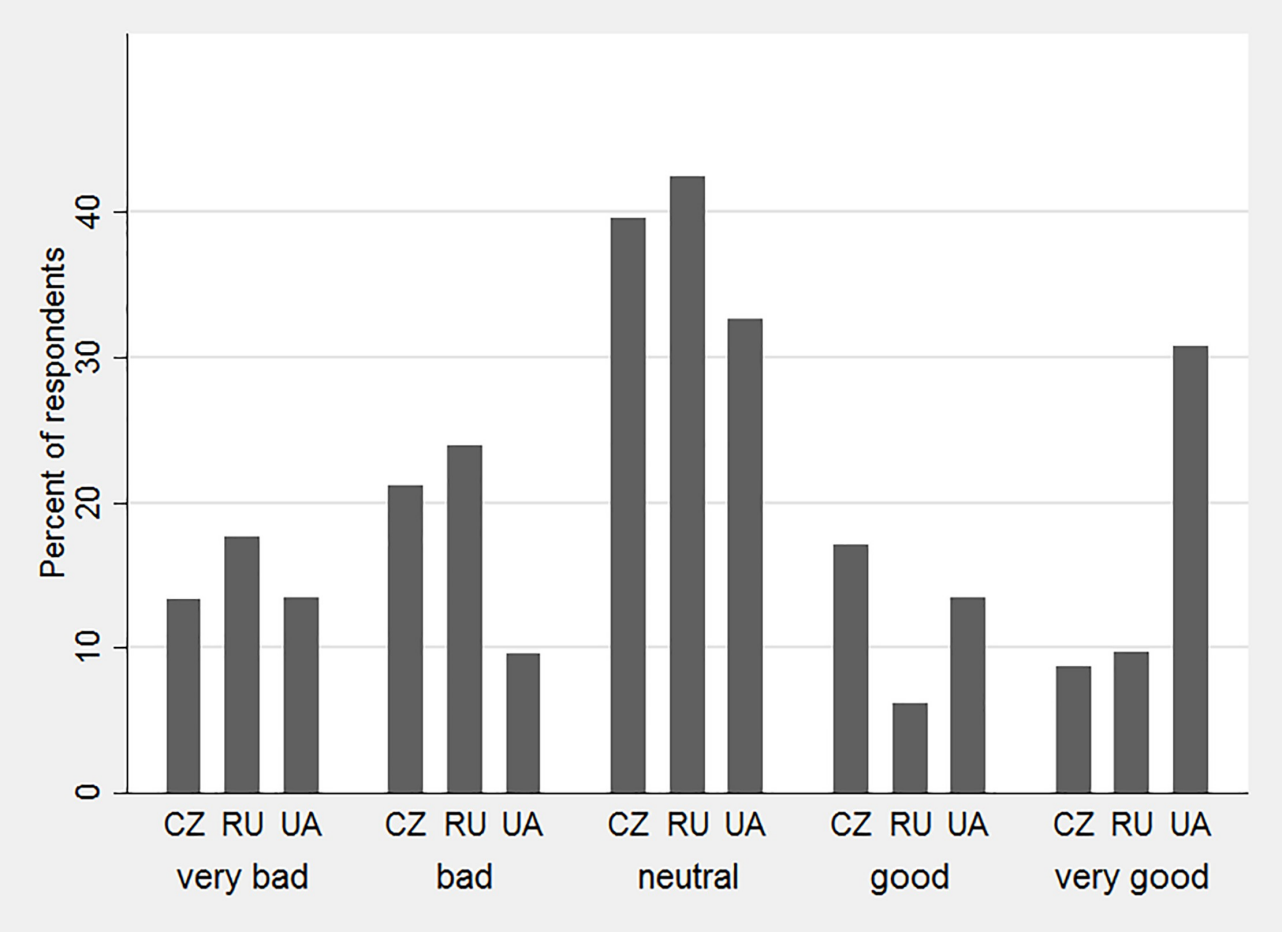
General attitude of young adults towards GMO crops by country (What do you think in general about GM crops? Production of GM crops is …).

The survey respondents' own general attitudes towards GMOs is illustrated in Fig 2, which shows how often each of the five approval categories was chosen by the young adults in the three countries. The approval levels in the samples from the three countries are spread over the full range of the five-point scale on attitudes, with 60% of the respondents stating a clear preference for or against GMOs (rather than a "neutral" approval level) in the Czech Republic and Russia and 70% in Ukraine. The class frequencies suggest that the respondents from Ukraine were, on average, more positive with regard to GM crops than the respondents from Russia and the Czech Republic.

Table 2 summarizes the dyadic data and reports how frequently the respondents classified their own and their fathers’, mothers’ and best friends’ GM food approval in each of the five categories. (The table covers less than the 382 participants included in the study since some of the respondents did not indicate the classification for both themselves and each individual referent person.) The largest counts are in the cells on the diagonals from upper left to lower right, which reflects a positive association of GM approval between YAs and their RPs on average over the three countries. The RPs of the vast majority of the YAs with positive attitudes towards GM crops also have positive attitudes, and vice versa. Low frequencies in the lower-left and upper-right corner cells show that few respondents (1.7%) report attitudes at the extreme opposite of their RPs. Even when collapsing the two negative and two positive approval categories, we observe only 8% of the respondents to be in discord with their RPs regarding their GM food attitude. In most of these discordant cases, the YA is positive and the RP is negative towards GM crops.

**Table 2:** Joint distribution of GM approval by young adults and their referent persons.

			GM approval level* of young adult					
GM approval level* of referent person (by type)			Very bad (1)	Bad (2)	Neutral (3)	Good (4)	Very good (5)	All appr. levels
	**Mother**							
Very bad (1)		Count	35	22	23	6	7	93
		*Percent*	*9*.*4*	*5*.*9*	*6*.*2*	*1*.*6*	*1*.*9*	*25*.*1*
Bad (2)		Count	6	33	34	7	2	82
		*Percent*	*1*.*6*	*8*.*9*	*9*.*2*	*1*.*9*	*0*.*5*	*22*.*1*
Neutral (3)		Count	10	19	80	20	14	143
		*Percent*	*2*.*7*	*5*.*1*	*21*.*6*	*5*.*4*	*3*.*8*	*38*.*5*
Good (4)		Count	4	2	9	13	9	37
		*Percent*	*1*.*1*	*0*.*5*	*2*.*4*	*3*.*5*	*2*.*4*	*10*
Very good (5)		Count	1	1	1	2	11	16
		*Percent*	*0*.*3*	*0*.*3*	*0*.*3*	*0*.*5*	*3*	*4*.*3*
All approval levels		Count	56	77	147	48	43	371
		*Percent*	*15*.*1*	*20*.*8*	*39*.*6*	*12*.*9*	*11*.*6*	100
	**Father**							
Very bad (1)		Count	36	29	29	7	7	108
		*Percent*	*9*.*5*	*7*.*7*	*7*.*7*	*1*.*8*	*1*.*8*	*28*.*5*
Bad (2)		Count	12	27	36	9	7	91
		*Percent*	*3*.*2*	*7*.*1*	*9*.*5*	*2*.*4*	*1*.*8*	*24*
Neutral (3)		Count	8	17	72	26	15	138
		*Percent*	*2*.*1*	*4*.*5*	*19*	*6*.*9*	*4*	*36*.*4*
Good (4)		Count	0	3	12	7	12	34
		*Percent*	*0*	*0*.*8*	*3*.*2*	*1*.*8*	*3*.*2*	*9*
Very good (5)		Count	0	2	1	0	5	8
		*Percent*	*0*	*0*.*5*	*0*.*3*	*0*	*1*.*3*	*2*.*1*
All approval levels		Count	56	78	150	49	46	379
		*Percent*	*14*.*8*	*20*.*6*	*39*.*6*	*12*.*9*	*12*.*1*	*100*
	**Best friend**							
Very bad (1)		Count	22	13	17	3	3	58
		*Percent*	*5*.*9*	*3*.*5*	*4*.*5*	*0*.*8*	*0*.*8*	*15*.*4*
Bad (2)		Count	16	35	34	8	5	98
		*Percent*	*4*.*3*	*9*.*3*	*9*	*2*.*1*	*1*.*3*	*26*.*1*
Neutral (3)		Count	15	27	82	22	12	158
		*Percent*	*4*	*7*.*2*	*21*.*8*	*5*.*9*	*3*.*2*	*42*
Good (4)		Count	1	2	13	15	11	42
		*Percent*	*0*.*3*	*0*.*5*	*3*.*5*	*4*	*2*.*9*	*11*.*2*
Very good (5)		Count	1	0	3	1	15	20
		*Percent*	*0*.*3*	*0*	*0*.*8*	*0*.*3*	*4*	*5*.*3*
All approval levels		Count	55	77	149	49	46	376
		Percent	*14*.*6*	*20*.*5*	*39*.*6*	*13*	*12*.*2*	*100*

* degree of GM food approval: response to survey item "What [do you/does your –] think about GM crops in general? GMO is Ȧ"

The pervasiveness of positive association is also reflected if we examine the differences in approval level within the dyads, i.e., between the YAs and their respective RPs. Table 3 presents the relative frequencies (in percent) of occurrence of such differences. For example, the number “22.9” in row "Mother" and column "1" indicates that the approval level of 22.9% of the respondents was one level above the GM food approval level of the respective respondents’ mothers. The highest frequencies we find are for exact concordance (43.3%), small deviations are also frequent, cases of YAs with substantially higher GM food approval compared with their RPs are rare, while the opposite case, substantially lower GM food approval of YAs than that of their RP are extremely rare. Looking at the differences between groups, we see that higher GM food approval of the YAs (compared to their RPs) is more prevalent regarding fathers than regarding mothers and friends. A higher GMO approval of YAs compared to their parents and friends is more frequent for young men than for young women.

**Table 3:** GMO approval levels of young adults compared with the approval levels of their referent persons.

	Deviation of young adults' GM food approval from their referent persons'									All deviations	Test of marginal homogeneity*
	-4	-3	-2	-1	0	1	2	3	4		
All cases	0.2	0.7	4	11.9	43.3	23.8	11.9	2.7	1.5	100	116.6(1) p < .00001
**Type of referent person**											
Mother	0.3	1.3	3.5	9.7	46.4	22.9	11.9	2.2	1.9	100	37.6(1) p < .00001
Father	.	0.5	3.2	10.8	38.8	27.2	14	3.7	1.8	100	73.9(1) p < .00001
Best friend	0.3	0.3	5.3	15.2	44.9	21.3	9.8	2.1	0.8	100	14.9(1) p = .00011
**Country**											
CZ	.	0.8	4.7	12.1	43.9	23.5	12.1	2.5	0.5	100	52.9(1) p < .00001
RU	0.3	0.3	2.1	12.5	42.9	26.5	11	2.4	2.1	100	50.0(1) p < .00001
UA	0.7	1.3	5.3	9.9	42.1	19.1	13.2	3.9	4.6	100	16.5 (1) p = .00005
**Gender**											
Male	.	0.5	4.4	11.2	38.8	24.7	13.7	4	2.8	100	71.4 (1) p < .00001
Female	0.3	0.9	3.7	12.4	46.1	23.3	10.8	1.9	0.7	100	48.0 (1) p < .00001

* We represent the joint distribution of cell counts *π*_*ij*_ for approval levels *i* and *j* and integer level scores *u*_*i*_. and *u*_*j*_ from 1 to 5 by ordinal quasi-symmetry models log(*π*_*ij*_/*π*_*ji*_) = *β*(*u*_*j*_−*u*_*i*_). Marginal homogeneity then implies that this model can be restricted to a symmetry model, *ß* = 0, which is the null in our likelihood ratio tests (column presents the LR statistic, DF, and p-value).

These differences in approval levels between YAs and RPs are statistically significant for all groups. The last column of Table 3 reports the results of tests of the marginal homogeneity hypothesis, which is equivalent to restricting ordinal quasi-symmetry models to symmetry [43]. Marginal homogeneity would have meant that the approval levels do not differ systematically between YAs and their RPs, and it is rejected for all groups. (Complete results of the quasi-symmetry and symmetry model estimations and LR tests are available in the supplementary S3 Appendix.)

### Loglinear model of associations

Despite GM food attitudes being significantly different between the YAs and their RPs, an inspection of the contingency tables suggests a positive association between YAs and RPs regarding their GMO attitudes. To formally test such an association and to measure the differences regarding the strength of the association across types of RP, countries, and gender, we use a loglinear model (2). Our initial maximum likelihood estimation does not fit the data very well. The standardized Pearson residuals of five observations exceed 4.0, which renders the model questionable even when considering the size of 450 cell counts. Despite the poor fit of the model, the estimate of 0.38 for *β* is significantly positive (Chi^2^(1) = 18.9, prob < .0001), asserting a positive association, while the estimates for all but one of the interaction terms of the L×L association with the explanatory variables ***R***, ***C***, and ***G*** ($\beta_{k}^{R},\beta_{l}^{C}$, and $\beta_{m}^{G}$) are not significantly different from zero. (The only exception was for the variable ***C***, as association differs to a statistically significant extent between young adults from the Czech Republic and those from Ukraine but not between the Czech Republic and Russia and not between Russia and Ukraine.) The results of this preliminary estimation are available in part C of the supplementary S3 Appendix. The poor fit is likely a consequence of the diverse structure of the sample described above. Descriptive tables suggested a pronounced positive association in the vast majority of the cases but a small group of cases with strongly discordant approval levels between YAs and their RPs. This heterogeneity, which we believe to correctly reflect the distribution of the population of interest, is so large that differences in association between these two groups dwarf the relatively subtle differences in terms of association between our explanatory variable levels, rendering their coefficient estimates statistically insignificant. To control for the effect of a small number of influential cases and to describe the patterns of association that characterize the vast majority of the sample, we concentrate on the bulk of the data, dropping some cases that differ most from the main cluster of cases. In particular, we drop seven cases (out of 382), selected based on standardized residuals, and repeat the estimations. The results on the parameters of particular interest for our study are presented in Table 4, while the complete estimation results are reported in part D of the supplementary S3 Appendix.

**Table 4:** Selected parameter estimates of the loglinear model of cell counts in the 5-way contingency table.

Effect	Level 1	Parameter	DF	Estimate	StdErr	lower 95% CI	upper 95% CI	ChiSq	P-val
									
yz		*β*	1	**0.498**	0.096	0.311	0.685	27.14	< .0001
yz*C	CZ	$\beta_{1}^{C}$	1	**0.154**	0.079	0.000	0.309	3.86	0.049
yz*C	RU	$\beta_{2}^{C}$	1	**0.206**	0.091	0.028	0.383	5.16	0.023
yz*C	UA	$\beta_{3}^{C}$	0	0	0.000	0.000	0.000		
yz*G	Male	$\beta_{1}^{G}$	1	**-0.135**	0.068	-0.269	-0.001	3.92	0.048
yz*G	Female	$\beta_{2}^{G}$	0	0	0.000	0.000	0.000		
yz*R	Best friend	$\beta_{1}^{R}$	1	**-0.166**	0.084	-0.330	-0.003	3.97	0.046
yz*R	Father	$\beta_{2}^{R}$	1	-0.132	0.086	-0.301	0.036	2.38	0.123
yz*R	Mother	$\beta_{3}^{R}$	0	0	0.000	0.000	0.000		

***yz***–linear-by-linear term of association (v^y^*v^***z***^ with v^***y***^ and *v*^***z***^ denoting *level scores*) between the GMO approval of 'young adult' and 'referent person'; ***R***–type of referent person; ***C***–country; ***G***—gender; Bold type indicates that the parameters are significantly different from zero.

We notice that the overall parameter estimate of the strength of association (0.498) is relatively similar to the estimate based on the complete data set (0.38). However, in this estimation, most of the interaction parameters measuring the differences in association between the levels of the covariates (***C***)ountry, (***G***)ender, and ***R*** (type of referent person) are significantly different (in a statistical sense with alpha = 5%) from the respective base levels. Additional Wald tests indicate that the differences between the non-base levels of the country variable (i.e., the difference between CZ and RU) and between the non-base levels of the type-of-referent-person variable (i.e., between fathers and best friends) are not statistically significant (Contrast Estimate Results are available in the supplementary S3 Appendix). The interaction parameter estimates confirm that an association regarding GM food attitudes between young adults and their referent persons is stronger in the Czech Republic and particularly in Russia than it is in Ukraine. Male young adults are less likely to have strong concordance in GMO attitudes with their referent persons than female young adults are. Finally, the association with mothers is significantly stronger than that with best friends, while the (positive) differences of mothers relative to fathers and fathers relative to best friends are not statistically significant.

From the parameter estimates, we derived (predicted) local odds ratios (ORs), which are interpretable in terms of the strength of the associations of GM food approval between YAs and RPs. As a consequence of the L×L specification, these ratios are homogeneous within each of the subtables for the combinations of covariates ***C***, ***G***, and ***R*** [43]. The local odds ratio for the *k*^th^ type of referent person, the *l*^th^ country, and gender *m* is computed as $LOR_{klm}=e^{\beta +\beta_{k}^{R}+\beta_{l}^{C}+\beta_{m}^{G}}$. For example, for Czech female YAs and their best friends, the $LOR_{klm}=e^{\beta +\beta_{1}^{R}+\beta_{1}^{C}+\beta_{2}^{G}}=e^{0.498-0.166+0.154+0}=1.63$. This means that the odds of a Czech female YA approving of GM crops by one category higher relative to a given degree of approval is 63% higher for each one-level-increase of her friend's GM food approval. Indeed, the estimated odds that a young adult assesses GM crops as "very good" (level 5) instead of "good" (level 4) is multiplied by 1.63 for each categorical increase in her friend's GM food approval. The same interpretation applies in the opposite direction, the friend's change in approval in response to increases of the young adult's approval. Table 5 presents the local ORs for all the combinations of covariate levels. (Confidence interval limits of local ORs were derived from the confidence limits for the predicted values of the mean.)

**Table 5:** Local odds ratios (*LOR*_*klm*_) as measures of the association of GM crop approval between young adults and their referent persons.

		Best friend		Father		Mother	
CZ	Male	1.42		1.47		1.68	
		1.33	1.52	1.38	1.57	1.54	1.83
	Female	1.63		1.68		1.92	
		1.52	1.74	1.57	1.80	1.77	2.09
RU	Male	1.50		1.55		1.77	
		1.38	1.63	1.43	1.68	1.60	1.96
	Female	1.71		1.77		2.02	
		1.57	1.88	1.62	1.94	1.82	2.25
UA	Male	1.22		1.26		1.44	
		1.15	1.30	1.19	1.34	1.32	1.58
	Female	1.39		1.44		1.65	
		1.29	1.51	1.33	1.57	1.50	1.82

the 95% confidence interval limits appear in small font.

The estimates of the confidence intervals of the local odds ratios are above one for all the groups defined by the combinations of levels of ***C***, ***G***, and ***R***, which indicates a significantly positive association. This means, that for all the groups represented in our analysis, a higher GM food approval is more likely with a higher GM food approval of referent persons. Differences between the groups exist, and they relate exclusively to the two-level interaction terms that describe the strength of the association conditional on the level of one explanatory variable. (Homogeneity of each explanatory variable's interaction effects with respect to a second explanatory variable was not rejected by LR tests.) The strongest association with referent persons was found between Russian female young adults and their mothers (local odds ratio = 2.02). Approval at one level higher by the mother doubles the probability of one level higher approval by the (adult) daughter, and vice versa. (We chose a loglinear model with two equally treated response variables to account for the fact that the influence between closely related (adult) persons typically goes in both directions. Nevertheless, an analysis of the relative strength of both directions could shed further light on the issue of the association of GMO attitudes.)

The weakest association is estimated between Ukrainian male young adults and their best friends (local OR = 1.22). Stronger GM food approval by a close friend still significantly increases the likelihood of a Ukrainian male young adult stating a higher GM food approval, but the point estimator of this increase in likelihood implies only a 22% increase in the local OR.

## Discussion and conclusions

In this study, we analyze the GM attitudes of young adults in three Central and Eastern European countries. As no similar studies have been conducted, our study is explorative, and the possibility of comparing our results with results from previous studies is very limited.

The descriptive statistics show that there are differences regarding GM approval between the three countries considered. The respondents from Ukraine were on average more positive with regard to GM crops than the respondents from the Czech Republic. The most negative attitude towards GM crops was expressed by the Russian respondents.

To answer the first of our research questions, we test whether there is an association between the GM food attitudes of young adults and the perceived attitudes of their parents and friends. The loglinear models of cell counts in contingency tables reveal a positive association between young adults and their referent individuals. The concordance in GM food attitudes with mothers is significantly stronger than concordance with best friends but not significantly different statistically from concordance with fathers.

The revealed associations can be interpreted in light of relationship theories as discussed by Simpson, Griskevicius, and Rothman [10], Wood and Hayes [12], and Bagozzi [8] and of Ajzen's theory of planned behavior. We assume that the perceived attitudes of referent persons reflect the social influences or pressures that a person is exposed to, that is, his or her normative beliefs or *subjective* (perception of social) *norms* using Ajzen's terminology. The associations we find between YAs’ and their RPs’ GM food attitudes hence suggest interconnections between the second and first determinants of behavioral intentions according to the theory, which are subjective norms and attitude, respectively. Regarding the theory of planned behavior, we can thus confirm the results of Tarkiainen and Sundqvist [25] because in our sample, attitudes and subjective norms are found not to be independent variables. Additionally, Fishbein and Ajzen [28] and Chang [29] find that subjective norms and attitudes may be directly linked. This link needs to be accounted for by appropriate interaction terms if unbiased estimates of the relative strength of the two determinants of behavioral intentions (attitudes and subjective norms) are desired.

Differences in association patterns between countries and between particular groups were revealed by loglinear analyses in our study. To answer the second research question, we test whether the association of GM food attitudes between young adults and their parents and peers differ across the three countries considered. Our results show that the association is stronger in Russia and the Czech Republic than it is in Ukraine. The study by Bagozzi, Wong, Abe, and Bergami [26] on the US, Italy, China and Japan suggested a stronger role of subjective norms in Eastern societies, with more interdependent self-concepts than in Western societies with more independent self-concepts. The cultural diversity between the countries in our study is not as large as that in the Bagozzi study, and it is unlikely that differences in the intradyad association of GM food attitudes between Russia and the Czech Republic on the one hand and Ukraine on the other hand correspond primarily to differing self-concepts in the three countries. More likely, there is a whole set of characteristics defining the dyadic context of each country that influences the degree of attitude concordance between young adults and their referent persons. Karney, Hops, Redding, Reis, Rothman, Simpson, et al. [44] emphasize that “a clear understanding of the dyadic context of behavior should determine which variables are most relevant for predicting behavior”, and this is likely to also apply to attitudes. The authors note that characteristics defining the dyadic context comprise personal traits (e.g., attachment orientation [45]), interpersonal constellations (trust, intimacy, satisfaction, commitment, communication, and power), and environmental characteristics (culture, social norms, and political environment). The characteristics are related to theories in relationship science, such as attachment theory, social norm models and motives for agreeing with others, as referred to by Simpson, Griskevicius, and Rothman [10] and Wood and Hayes [12]. Country-specific cultural patterns regarding typical intergenerational relations, gender roles, and power relationships in families are possible determinants of attitude association among referent persons in a country. However, insights on these links are beyond the scope of our study, and information that can reliably reflect the respective psychological constructs is not available in our data. These factors form, however, a promising area for further study.

Regarding the third research question, we tested whether the association of GM crop attitudes between young adults and their parents and peers differed by gender. The association between the attitudes of female young adults and their referent persons was found to be stronger than between male young adults and their referent individuals. The odds ratios derived from our estimates indicate that the strongest association of GM food attitudes exists between Russian and Czech female young adults and their mothers. However, we cannot rule out that association of attitudes is partially caused by external factors that independently influence both referent persons in similar ways, and we are not able to conclude from this research who affects who’s GM food attitudes. That is, do young females influence the attitudes of their mothers, is it the other way around, or is the influence mutual? The direction of influence between referent persons’ GM food attitudes is likely to depend on one’s knowledge of GMOs and the perceived knowledge of the referent person, as the *deficit model* suggests. This model explains the disapproval of a technology with one’s insufficient knowledge about that technology [46, 47]. It has found only weak empirical support in the case of GMOs, potentially due to a lack of reliable assessments of GMO knowledge, but recent progress towards such an assessment will open new opportunities here [48].

The weakest associations are estimated between Ukrainian male young adults and their fathers and best friends. These results suggest a possible tendency of males, particularly in Ukraine, to develop their attitudes relatively independently from their referent individuals. Possible hypotheses as explanations are that males may be less anxiously or more avoidantly attached (in the sense of attachment theory) than women or that males typically differ from women regarding the relationship norms that they endorse [49, 50]. Another possible interpretation of this finding is that young male adults may form their attitudes with less mutual exchange within family circles and among peers than females do. Testing to what extent such hypotheses from various strands of relationship science can explain differences in associations of GM food attitudes would be a logical next step towards understanding the underlying interpersonal processes, as suggested by Bagozzi [8], for future research.

Our results have some generalizable implications. Many European food consumers have critical attitudes towards GMO foods; they are aloof and expect governments to restrict the development of GMO food crop varieties and introduce mandatory GMO labeling or restrictions on marketing. Such a critical attitude may diminish over time, as we found that the GMO attitudes of young adults are more positive than their parents’. However, a strong and consistent positive association will possibly slow any change in the attitudes of those “pioneers” changing their own values or assessment relatively quickly (e.g., due to new insights they gain) as their attitudes are also connected to those of their referent persons. At the same time, the changed attitudes of some “pioneers” are likely to pull their referent persons in the same direction, as the strong positive association suggests. This means that marketing activities narrowly focused on particular population strata, for example, young adults, will also influence the groups of referent persons of the target group, and the referent groups’ inertance will dampen the effects on the target groups. (However, this effect depends on the assumption that there is mutual influence on each other’s attitudes among referent persons.) These considerations are relevant for the marketing activities of food firms as well as for policy, as political support of technological developments must account for public attitudes and the acceptance of technologies. They should be appreciated when planning communication strategies, marketing campaigns, etc. in the field. This can have significant implications on the market potential of GM and non-GM foods, the diffusion of agricultural technologies and consequently on the development of the agrifood economy and competitive positions in international agricultural and food trade.

Our study has implications and suggests potential directions for further research. On the one hand, the confirmation of the result, that attitude is not independent from subjective norms, is methodologically relevant for studies assessing the relative impact of both of these groups of behavioral determinants. On the other hand, the differences in attitude association between countries, gender and type of referent person motivate further research. Future studies may use concepts of personal traits (such as attachment orientation and self-concepts) and communication patterns as well as culture and power relationships to analyze the impact of referent persons on attitude formation. They may integrate the contribution of other social sciences (namely, sociology and anthropology). Rigorous qualitative research could aid in the exploration of variables that explain the perception of attitudes towards GMOs. Major aspects of the context of living that could not be addressed in this study should be accounted for.

A major limitation of our explorative study is that our sample is nonrandom and cannot be assumed to be fully representative of the population of interest. Future studies with larger random samples are needed to verify and extend the results and compare them with attitude associations regarding technologies and food types other than GMO foods.

In this research, we analyze the GM food attitudes of young adults and their referent persons as perceived by young adults. A promising modification for future research would be to consider GM food approval stated (or revealed) by the referent persons themselves rather than the attitudes perceived by others (the respondents). Moreover, a promising improvement would be to represent the construct of GM food approval by a scale based on several items so that the reliability of that scale can be assessed. This is not possible for our representation of GM food approval by a single item. The impact of other covariates that are likely to influence GM food attitudes as well as the association of GM food attitudes between referent persons could not be assessed in this study due to limitations regarding the number of factors that can be accommodated with our methodological approach. This is reflected in the fact that a clear image of behavioral patterns only emerged after excluding a (small) number of observations. Future research should try to identify characteristics that distinguish this small group from the majority, for example, by sociodemographic factors such as family structure or education.

## Supporting information

S1 Appendix Survey questionnaire. (PDF)

S2 Appendix Contingency table. (PDF)

S3 Appendix Intermediate estimation results. (PDF)
