# Supplementary material for: Association of attitudes towards genetically modified food among young adults and their referent persons
Source: PLoS One. 2019 Feb 4;14(2):e0211879. doi: 10.1371/journal.pone.0211879 (PMC6361467; doi:10.1371/journal.pone.0211879)
Supplement: S1 Appendix — (PDF) [file pone.0211879.s001.pdf]

Association of attitudes towards genetically modified food among young adults and their referent persons

Stephan Brosig and Miroslava Bavorova

S1 Appendix: Survey questionnaire

Czech University of Life Sciences
Faculty of Tropical Agri Science

GM food opinion survey

We are students of a course in world economy and agriculture at the Czech University of Life Sciences in Prague. We conduct a survey about the opinions of people regarding GMO (genetically modified organisms) in agricultural and food production. We would like to ask you to fill the questionnaire and to help to make our investigation more realistic. This short questionnaire about GM crops and food just takes you about five minutes. All collected data are anonymous and will be kept confidential and we will only publish aggregated results. We would be grateful for your opinion and time!

1. How often do you hear news about genetically modified organism?

1. Very often	2	3	4	5. Never
❍	❍	❍	❍	❍

2. What do you think in general about GM crops? Production of GM crops is..

1. Very good	2	3	4	5. Very bad
❍	❍	❍	❍	❍

3 In which country do you spent most time?

----

4. Do you think the cultivation of GM crops is risky for nature?

1. Very risky	2	3	4	5. Not risky at all
❍	❍	❍	❍	❍

5. Do you think the food from GM crops is risky for peoples' health?

1. Very risky	2	3	4	5. Not risky at all
❍	❍	❍	❍	❍

6. Do you agree that GM crops bring significant economic benefits for farmers in developing countries?

1. Fully agree	2	3	4	5. Completely disagree
❍	❍	❍	❍	❍

7. Do you agree that production of more GM crops will lower the food prices for consumers in developed countries?

1. Fully agree	2	3	4	5. Completely disagree
❍	❍	❍	❍	❍

8. If possible, please assess what your father thinks in general about GM crops? GM crop production is...

1. Very good	2	3	4	5. Very bad
❍	❍	❍	❍	❍

9. If possible, please assess what your mother thinks in general about GM crops? GM crop production is...

1. Very good	2	3	4	5. Very bad
❍	❍	❍	❍	❍

10. If possible, please assess what your best friend thinks in general about GM crops? GM crop production is....

1. Very good	2	3	4	5. Very bad
❍	❍	❍	❍	❍

11. What is your age?

Under 18	18-30	Over 30
❍	❍	❍

12. What is your nationality?

----

13. What is your gender?

----

14. What is your highest educational grade achieved? (only in Czech survey)

----

15. How well are you informed about GM crop and food production?

1. Very well	2	3	4	5. Very little
❍	❍	❍	❍	❍

16. If possible, please assess how well your father is informed about GM crop and food production?

1. Very well	2	3	4	5. Very little
❍	❍	❍	❍	❍

17. If possible, please assess how well your mother is informed about GM crop and food production?

1. Very well	2	3	4	5. Very little
❍	❍	❍	❍	❍

18. If possible, please assess how well your best friend is informed about GM crops production?

1. Very well	2	3	4	5. Very little
❍	❍	❍	❍	❍

19. Did you ever study agriculture or food sciences or work in that field?

Yes	No
❍	❍

20. Did your father ever study agriculture or food sciences or work in that field?

Yes	No
❍	❍

21. Did your mother ever study agriculture or food sciences or work in that field?

Yes	No
❍	❍

Thank you for your support!
