# Supplementary material for: Association of attitudes towards genetically modified food among young adults and their referent persons
Source: PLoS One. 2019 Feb 4;14(2):e0211879. doi: 10.1371/journal.pone.0211879 (PMC6361467; doi:10.1371/journal.pone.0211879)
Supplement: S2 Appendix — (PDF) [file pone.0211879.s002.pdf]

Association of attitudes towards genetically modified food among young adults and their referent persons

Stephan Brosig and Miroslava Bavorova

S2 Appendix: Contingency table

Table A: The complete 5-way contingency table (as stylized in Figure 1) analyzed with loglinear model......... 1

Table B: Summary of cell counts in 5-way contingency table... 2

Table A: The complete 5-way contingency table (as stylized in Figure 1) analyzed with loglinear model

Cell counts			Gender: 1_Male, rating of young adult					Gender: 2_Female, rating of young adult				
type of referent pers	Country	rating of referent pers	1_Very bad	2_Bad	3_Neutral	4_Good	5_Very good	1_Very bad	2_Bad	3_Neutral	4_Good	5_Very good
1_BFriend	1_CZ	1_Very bad	4	.	.	2	1	8	8	9	1	.
		2_Bad	1	7	5	3	.	3	15	13	3	1
		3_Neutral	3	5	14	7	7	6	8	35	6	3
		4_Good	1	1	3	3	1	3	1	5	8	2
		5_Very good	.	.	.	1	1	.	.	1	.	2
	2_RU	1_Very bad	4	3	4	.	2	14	10	5	2	1
		2_Bad	1	3	6	.	1	1	5	8	1	.
		3_Neutral	.	2	8	2	1	.	4	14	1	1
		4_Good	.	.	.	1	2	.	.	1	.	.
		5_Very good	.	.	.	.	1	.	.	.	.	1
	3_UA	1_Very bad	2	1	2	.	3	3	.	3	1	.
		2_Bad	.	2	1	.	.	.	1	1	.	.
		3_Neutral	.	.	6	3	1	1	.	3	1	1
		4_Good	.	.	.	1	3	.	.	.	.	1
		5_Very good	.	.	.	1	5	1	1	.	.	1
2_Father	1_CZ	1_Very bad	4	5	2	3	.	11	11	13	2	1
		2_Bad	2	6	6	4	2	6	13	13	3	1
		3_Neutral	3	2	11	9	8	3	7	33	10	2
		4_Good	.	.	3	.	1	.	1	4	5	4
		5_Very good	.	.	.	.	.	.	1	.	.	.
	2_RU	1_Very bad	4	2	4	.	2	14	10	7	1	1
		2_Bad	1	2	7	1	3	1	5	9	1	.
		3_Neutral	.	2	7	2	.	.	4	11	2	2
		4_Good	.	1	1	.	2	.	.	2	.	.
		5_Very good	.	1	.	.	.	.	.	.	.	1
	3_UA	1_Very bad	1	1	1	1	3	2	.	2	.	.
		2_Bad	1	.	1	.	1	1	1	.	.	.
		3_Neutral	.	1	4	2	2	2	1	6	1	1
		4_Good	.	1	2	2	4	.	.	.	.	1
		5_Very good	.	.	1	.	3	.	.	.	.	1
3_Mother	1_CZ	1_Very bad	4	1	3	1	.	8	5	6	1	1
		2_Bad	1	5	4	2	1	7	21	17	4	1
		3_Neutral	4	5	12	9	2	4	7	34	6	3
		4_Good	.	1	2	4	5	.	.	5	8	2
		5_Very good	.	.	1	.	3	.	.	1	.	1
	2_RU	1_Very bad	3	3	1	.	.	5	3	3	.	1
		2_Bad	1	1	1	1	1	6	7	11	.	.
		3_Neutral	1	4	16	2	4	3	8	12	2	1
		4_Good	.	.	.	.	.	.	1	3	2	1
		5_Very good	.	.	1	.	2	1	.	.	.	1
	3_UA	1_Very bad	.	1	3	1	1	2	.	1	.	.
		2_Bad	1	.	.	1	.	.	1	1	.	2
		3_Neutral	1	2	3	1	2	2	1	5	2	.
		4_Good	.	.	2	1	3	1	.	1	.	.
		5_Very good	.	.	.	1	7	.	.	.	.	1

Table B: Summary of cell counts in 5-way contingency table

Cell counts		Gender		
		1_Male	2_Fem-ale	All
type of referent pers	Country			
1_BFriend	1_CZ	70	141	211
	2_RU	41	69	110
	3_UA	31	19	50
	All	142	229	371
2_Father	Country			
	1_CZ	71	144	215
	2_RU	42	71	113
	3_UA	32	19	51
	All	145	234	379
3_Mother	Country			
	1_CZ	70	142	212
	2_RU	42	71	113
	3_UA	31	20	51
	All	143	233	376
All		430	696	1126
