# Supplementary material for: Association of attitudes towards genetically modified food among young adults and their referent persons
Source: PLoS One. 2019 Feb 4;14(2):e0211879. doi: 10.1371/journal.pone.0211879 (PMC6361467; doi:10.1371/journal.pone.0211879)
Supplement: S3 Appendix — (PDF) [file pone.0211879.s003.pdf]

Association of attitudes towards genetically modified food among young adults and their referent persons

Stephan Brosig and Miroslava Bavorova

S3 Appendix: Intermediate estimation results

Contents

Part A: Detailed marginal homogeneity test results 2

Part B: Summary of marginal homogeneity test results 20

Part C: Loglinear model estimation of equation (2) using full data set........ 21

Part D: Final loglinear model estimation of equation (2) as excerpted in Table 4........ 24

Part A: Detailed marginal homogeneity test results

Tests of marginal homogeneity for complete data and subsets: Testing H0: symmetry against H1: ordered quasi-symmetry. (Agresti, Analysis of Ordinal Categorical Data, 2nd Ed. Section 8.4.7)

```
Symmetry, subset: R>0 (all data)

The GENMOD Procedure

                             Model Information

Data Set                      WORK.GMOCTALLOBS1
Distribution                           Binomial
Link Function                             Logit
Response Variable (Events)              countYA    Frequency Count
Response Variable (Trials)                 nges


Number of Observations Read          10
Number of Observations Used          10
Number of Events                    449
Number of Trials                    638


         Response Profile

 Ordered    Binary          Total
   Value    Outcome     Frequency

       1    Event             449
       2    Nonevent          189


             Criteria For Assessing Goodness Of Fit

Criterion                     DF           Value        Value/DF

Deviance                      10        141.4182         14.1418
Scaled Deviance               10        141.4182         14.1418
Pearson Chi-Square            10        129.5355         12.9535
Scaled Pearson X2             10        129.5355         12.9535
Log Likelihood                         -442.2279
Full Log Likelihood                     -90.0615
AIC (smaller is better)                 180.1230
AICC (smaller is better)                180.1230
BIC (smaller is better)                 180.1230


Algorithm converged.


                    Analysis Of Maximum Likelihood Parameter Estimates

                            Standard     Wald 95% Confidence          Wald
Parameter    DF    Estimate       Error          Limits          Chi-Square    Pr > ChiSq

Intercept     0      0.0000      0.0000      0.0000      0.0000           .             .
Scale         0      1.0000      0.0000      1.0000      1.0000

NOTE: The scale parameter was held fixed.


    Lagrange Multiplier Statistics

Parameter     Chi-Square    Pr > ChiSq

Intercept       105.9561        <.0001
```

Ordinal Quasi Symmetry model, subset: R>0 (all data)

The GENMOD Procedure

Model Information

Data Set	WORK.GMOCTALLOBS1	
Distribution	Binomial	
Link Function	Logit	
Response Variable (Events)	countYA	Frequency Count
Response Variable (Trials)	nges	

Number of Observations Read	10
Number of Observations Used	10
Number of Events	449
Number of Trials	638

Response Profile

Ordered Value	Binary Outcome	Total Frequency
1	Event	449
2	Nonevent	189

Criteria For Assessing Goodness Of Fit

Criterion	DF	Value	Value/DF
Deviance	9	24.8502	2.7611
Scaled Deviance	9	24.8502	2.7611
Pearson Chi-Square	9	21.6118	2.4013
Scaled Pearson X2	9	21.6118	2.4013
Log Likelihood		-383.9439	
Full Log Likelihood		-31.7775	
AIC (smaller is better)		65.5550	
AICC (smaller is better)		66.0550	
BIC (smaller is better)		65.8576	

Algorithm converged.

Analysis Of Maximum Likelihood Parameter Estimates

Parameter	DF	Estimate	Standard Error	Wald 95% Confidence Limits		Wald Chi-Square	Pr > ChiSq
Intercept	0	0.0000	0.0000	0.0000	0.0000	.	.
score	1	0.5716	0.0584	0.4573	0.6860	95.94	<.0001
Scale	0	1.0000	0.0000	1.0000	1.0000		

NOTE: The scale parameter was held fixed.

Lagrange Multiplier Statistics

Parameter	Chi-Square	Pr > ChiSq
Intercept	2.1170	0.1457

Symmetry, subset: R=1

The GENMOD Procedure

Model Information

Data Set	WORK.GMOCTALLOBS1	
Distribution	Binomial	
Link Function	Logit	
Response Variable (Events)	countYA	Frequency Count
Response Variable (Trials)	nges	

Number of Observations Read	10
Number of Observations Used	10
Number of Events	144
Number of Trials	199

Response Profile

Ordered Value	Binary Outcome	Total Frequency
1	Event	144
2	Nonevent	55

Criteria For Assessing Goodness Of Fit

Criterion	DF	Value	Value/DF
Deviance	10	50.5762	5.0576
Scaled Deviance	10	50.5762	5.0576
Pearson Chi-Square	10	46.4141	4.6414
Scaled Pearson X2	10	46.4141	4.6414
Log Likelihood		-137.9363	
Full Log Likelihood		-39.3995	
AIC (smaller is better)		78.7990	
AICC (smaller is better)		78.7990	
BIC (smaller is better)		78.7990	

Algorithm converged.

Analysis Of Maximum Likelihood Parameter Estimates

Parameter	DF	Estimate	Standard Error	Wald 95% Confidence Limits		Wald Chi-Square	Pr > ChiSq
Intercept	0	0.0000	0.0000	0.0000	0.0000	.	.
Scale	0	1.0000	0.0000	1.0000	1.0000		

NOTE: The scale parameter was held fixed.

Lagrange Multiplier Statistics

Parameter	Chi-Square	Pr > ChiSq
Intercept	39.8040	<.0001

Ordinal Quasi Symmetry model, subset: R=1

The GENMOD Procedure

Model Information

Data Set	WORK.GMOCTALLOBS1	
Distribution	Binomial	
Link Function	Logit	
Response Variable (Events)	countYA	Frequency Count
Response Variable (Trials)	nges	

Number of Observations Read	10
Number of Observations Used	10
Number of Events	144
Number of Trials	199

Response Profile

Ordered Value	Binary Outcome	Total Frequency
1	Event	144
2	Nonevent	55

Criteria For Assessing Goodness Of Fit

Criterion	DF	Value	Value/DF
Deviance	9	13.0227	1.4470
Scaled Deviance	9	13.0227	1.4470
Pearson Chi-Square	9	13.1072	1.4564
Scaled Pearson X2	9	13.1072	1.4564
Log Likelihood		-119.1596	
Full Log Likelihood		-20.6228	
AIC (smaller is better)		43.2456	
AICC (smaller is better)		43.7456	
BIC (smaller is better)		43.5482	

Algorithm converged.

Analysis Of Maximum Likelihood Parameter Estimates

Parameter	DF	Estimate	Standard Error	Wald 95% Confidence Limits		Wald Chi-Square	Pr > ChiSq
Intercept	0	0.0000	0.0000	0.0000	0.0000	.	.
score	1	0.5625	0.1017	0.3631	0.7619	30.57	<.0001
Scale	0	1.0000	0.0000	1.0000	1.0000		

NOTE: The scale parameter was held fixed.

Lagrange Multiplier Statistics

Parameter	Chi-Square	Pr > ChiSq
Intercept	4.4010	0.0359

Symmetry, subset: R=2

The GENMOD Procedure

Model Information

Data Set	WORK.GMOCTALLOBS1	
Distribution	Binomial	
Link Function	Logit	
Response Variable (Events)	countYA	Frequency Count
Response Variable (Trials)	nges	

Number of Observations Read	10
Number of Observations Used	10
Number of Events	177
Number of Trials	232

Response Profile

Ordered Value	Binary Outcome	Total Frequency
1	Event	177
2	Nonevent	55

Criteria For Assessing Goodness Of Fit

Criterion	DF	Value	Value/DF
Deviance	10	88.9964	8.8996
Scaled Deviance	10	88.9964	8.8996
Pearson Chi-Square	10	74.9647	7.4965
Scaled Pearson X2	10	74.9647	7.4965
Log Likelihood		-160.8101	
Full Log Likelihood		-55.9758	
AIC (smaller is better)		111.9517	
AICC (smaller is better)		111.9517	
BIC (smaller is better)		111.9517	

Algorithm converged.

Analysis Of Maximum Likelihood Parameter Estimates

Parameter	DF	Estimate	Standard Error	Wald 95% Confidence Limits		Wald Chi-Square	Pr > ChiSq
Intercept	0	0.0000	0.0000	0.0000	0.0000	.	.
Scale	0	1.0000	0.0000	1.0000	1.0000		

NOTE: The scale parameter was held fixed.

Lagrange Multiplier Statistics

Parameter	Chi-Square	Pr > ChiSq
Intercept	64.1552	<.0001

Ordinal Quasi Symmetry model, subset: R=2

The GENMOD Procedure

Model Information

Data Set	WORK.GMOCTALLOBS1	
Distribution	Binomial	
Link Function	Logit	
Response Variable (Events)	countYA	Frequency Count
Response Variable (Trials)	nges	

Number of Observations Read	10
Number of Observations Used	10
Number of Events	177
Number of Trials	232

Response Profile

Ordered Value	Binary Outcome	Total Frequency
1	Event	177
2	Nonevent	55

Criteria For Assessing Goodness Of Fit

Criterion	DF	Value	Value/DF
Deviance	9	15.0944	1.6772
Scaled Deviance	9	15.0944	1.6772
Pearson Chi-Square	9	11.2460	1.2496
Scaled Pearson X2	9	11.2460	1.2496
Log Likelihood		-123.8591	
Full Log Likelihood		-19.0248	
AIC (smaller is better)		40.0496	
AICC (smaller is better)		40.5496	
BIC (smaller is better)		40.3522	

Algorithm converged.

Analysis Of Maximum Likelihood Parameter Estimates

Parameter	DF	Estimate	Standard Error	Wald 95% Confidence Limits		Wald Chi-Square	Pr > ChiSq
Intercept	0	0.0000	0.0000	0.0000	0.0000	.	.
score	1	0.8070	0.1107	0.5900	1.0239	53.15	<.0001
Scale	0	1.0000	0.0000	1.0000	1.0000		

NOTE: The scale parameter was held fixed.

Lagrange Multiplier Statistics

Parameter	Chi-Square	Pr > ChiSq
Intercept	0.5106	0.4749

Symmetry, subset: R=3

The GENMOD Procedure

Model Information

Data Set	WORK.GMOCTALLOBS1	
Distribution	Binomial	
Link Function	Logit	
Response Variable (Events)	countYA	Frequency Count
Response Variable (Trials)	nges	

Number of Observations Read	10
Number of Observations Used	10
Number of Events	128
Number of Trials	207

Response Profile

Ordered Value	Binary Outcome	Total Frequency
1	Event	128
2	Nonevent	79

Criteria For Assessing Goodness Of Fit

Criterion	DF	Value	Value/DF
Deviance	10	31.9947	3.1995
Scaled Deviance	10	31.9947	3.1995
Pearson Chi-Square	10	27.8862	2.7886
Scaled Pearson X2	10	27.8862	2.7886
Log Likelihood		-143.4815	
Full Log Likelihood		-29.3969	
AIC (smaller is better)		58.7937	
AICC (smaller is better)		58.7937	
BIC (smaller is better)		58.7937	

Algorithm converged.

Analysis Of Maximum Likelihood Parameter Estimates

Parameter	DF	Estimate	Standard Error	Wald 95% Confidence Limits		Wald Chi-Square	Pr > ChiSq
Intercept	0	0.0000	0.0000	0.0000	0.0000	.	.
Scale	0	1.0000	0.0000	1.0000	1.0000		

NOTE: The scale parameter was held fixed.

Lagrange Multiplier Statistics

Parameter	Chi-Square	Pr > ChiSq
Intercept	11.5990	0.0007

Ordinal Quasi Symmetry model, subset: R=3

The GENMOD Procedure

Model Information

Data Set	WORK.GMOCTALLOBS1	
Distribution	Binomial	
Link Function	Logit	
Response Variable (Events)	countYA	Frequency Count
Response Variable (Trials)	nges	

Number of Observations Read	10
Number of Observations Used	10
Number of Events	128
Number of Trials	207

Response Profile

Ordered Value	Binary Outcome	Total Frequency
1	Event	128
2	Nonevent	79

Criteria For Assessing Goodness Of Fit

Criterion	DF	Value	Value/DF
Deviance	9	17.1025	1.9003
Scaled Deviance	9	17.1025	1.9003
Pearson Chi-Square	9	14.6723	1.6303
Scaled Pearson X2	9	14.6723	1.6303
Log Likelihood		-136.0354	
Full Log Likelihood		-21.9508	
AIC (smaller is better)		45.9016	
AICC (smaller is better)		46.4016	
BIC (smaller is better)		46.2041	

Algorithm converged.

Analysis Of Maximum Likelihood Parameter Estimates

Parameter	DF	Estimate	Standard Error	Wald 95% Confidence Limits		Wald Chi-Square	Pr > ChiSq
Intercept	0	0.0000	0.0000	0.0000	0.0000	.	.
score	1	0.3557	0.0958	0.1680	0.5435	13.79	0.0002
Scale	0	1.0000	0.0000	1.0000	1.0000		

NOTE: The scale parameter was held fixed.

Lagrange Multiplier Statistics

Parameter	Chi-Square	Pr > ChiSq
Intercept	0.0614	0.8043

Symmetry, subset: C=1

The GENMOD Procedure

Model Information

Data Set	WORK.GMOCTALLOBS1	
Distribution	Binomial	
Link Function	Logit	
Response Variable (Events)	countYA	Frequency Count
Response Variable (Trials)	nges	

Number of Observations Read	10
Number of Observations Used	10
Number of Events	246
Number of Trials	358

Response Profile

Ordered Value	Binary Outcome	Total Frequency
1	Event	246
2	Nonevent	112

Criteria For Assessing Goodness Of Fit

Criterion	DF	Value	Value/DF
Deviance	10	75.2667	7.5267
Scaled Deviance	10	75.2667	7.5267
Pearson Chi-Square	10	67.5657	6.7566
Scaled Pearson X2	10	67.5657	6.7566
Log Likelihood		-248.1467	
Full Log Likelihood		-53.0959	
AIC (smaller is better)		106.1919	
AICC (smaller is better)		106.1919	
BIC (smaller is better)		106.1919	

Algorithm converged.

Analysis Of Maximum Likelihood Parameter Estimates

Parameter	DF	Estimate	Standard Error	Wald 95% Confidence Limits		Wald Chi-Square	Pr > ChiSq
Intercept	0	0.0000	0.0000	0.0000	0.0000	.	.
Scale	0	1.0000	0.0000	1.0000	1.0000		

NOTE: The scale parameter was held fixed.

Lagrange Multiplier Statistics

Parameter	Chi-Square	Pr > ChiSq
Intercept	50.1564	<.0001

Ordinal Quasi Symmetry model, subset: C=1

The GENMOD Procedure

Model Information

Data Set	WORK.GMOCTALLOBS1	
Distribution	Binomial	
Link Function	Logit	
Response Variable (Events)	countYA	Frequency Count
Response Variable (Trials)	nges	

Number of Observations Read	10
Number of Observations Used	10
Number of Events	246
Number of Trials	358

Response Profile

Ordered Value	Binary Outcome	Total Frequency
1	Event	246
2	Nonevent	112

Criteria For Assessing Goodness Of Fit

Criterion	DF	Value	Value/DF
Deviance	9	22.3601	2.4845
Scaled Deviance	9	22.3601	2.4845
Pearson Chi-Square	9	20.1737	2.2415
Scaled Pearson X2	9	20.1737	2.2415
Log Likelihood		-221.6934	
Full Log Likelihood		-26.6427	
AIC (smaller is better)		55.2853	
AICC (smaller is better)		55.7853	
BIC (smaller is better)		55.5879	

Algorithm converged.

Analysis Of Maximum Likelihood Parameter Estimates

Parameter	DF	Estimate	Standard Error	Wald 95% Confidence Limits		Wald Chi-Square	Pr > ChiSq
Intercept	0	0.0000	0.0000	0.0000	0.0000	.	.
score	1	0.5248	0.0774	0.3730	0.6765	45.92	<.0001
Scale	0	1.0000	0.0000	1.0000	1.0000		

NOTE: The scale parameter was held fixed.

Lagrange Multiplier Statistics

Parameter	Chi-Square	Pr > ChiSq
Intercept	1.4132	0.2345

Symmetry, subset: C=2

The GENMOD Procedure

Model Information

Data Set	WORK.GMOCTALLOBS1	
Distribution	Binomial	
Link Function	Logit	
Response Variable (Events)	countYA	Frequency Count
Response Variable (Trials)	nges	

Number of Observations Read	10
Number of Observations Used	10
Number of Events	141
Number of Trials	192

Response Profile

Ordered Value	Binary Outcome	Total Frequency
1	Event	141
2	Nonevent	51

Criteria For Assessing Goodness Of Fit

Criterion	DF	Value	Value/DF
Deviance	10	58.7432	5.8743
Scaled Deviance	10	58.7432	5.8743
Pearson Chi-Square	10	51.8408	5.1841
Scaled Pearson X2	10	51.8408	5.1841
Log Likelihood		-133.0843	
Full Log Likelihood		-40.7545	
AIC (smaller is better)		81.5090	
AICC (smaller is better)		81.5090	
BIC (smaller is better)		81.5090	

Algorithm converged.

Analysis Of Maximum Likelihood Parameter Estimates

Parameter	DF	Estimate	Standard Error	Wald 95% Confidence Limits		Wald Chi-Square	Pr > ChiSq
Intercept	0	0.0000	0.0000	0.0000	0.0000	.	.
Scale	0	1.0000	0.0000	1.0000	1.0000		

NOTE: The scale parameter was held fixed.

Lagrange Multiplier Statistics

Parameter	Chi-Square	Pr > ChiSq
Intercept	42.1875	<.0001

Ordinal Quasi Symmetry model, subset: C=2

The GENMOD Procedure

Model Information

Data Set	WORK.GMOCTALLOBS1	
Distribution	Binomial	
Link Function	Logit	
Response Variable (Events)	countYA	Frequency Count
Response Variable (Trials)	nges	

Number of Observations Read	10
Number of Observations Used	10
Number of Events	141
Number of Trials	192

Response Profile

Ordered Value	Binary Outcome	Total Frequency
1	Event	141
2	Nonevent	51

Criteria For Assessing Goodness Of Fit

Criterion	DF	Value	Value/DF
Deviance	9	8.7303	0.9700
Scaled Deviance	9	8.7303	0.9700
Pearson Chi-Square	9	7.2534	0.8059
Scaled Pearson X2	9	7.2534	0.8059
Log Likelihood		-108.0778	
Full Log Likelihood		-15.7481	
AIC (smaller is better)		33.4961	
AICC (smaller is better)		33.9961	
BIC (smaller is better)		33.7987	

Algorithm converged.

Analysis Of Maximum Likelihood Parameter Estimates

Parameter	DF	Estimate	Standard Error	Wald 95% Confidence Limits		Wald Chi-Square	Pr > ChiSq
Intercept	0	0.0000	0.0000	0.0000	0.0000	.	.
score	1	0.7379	0.1208	0.5011	0.9746	37.33	<.0001
Scale	0	1.0000	0.0000	1.0000	1.0000		

NOTE: The scale parameter was held fixed.

Lagrange Multiplier Statistics

Parameter	Chi-Square	Pr > ChiSq
Intercept	0.1242	0.7245

Symmetry, subset: C=3

The GENMOD Procedure

Model Information

Data Set	WORK.GMOCTALLOBS1	
Distribution	Binomial	
Link Function	Logit	
Response Variable (Events)	countYA	Frequency Count
Response Variable (Trials)	nges	

Number of Observations Read	10
Number of Observations Used	10
Number of Events	62
Number of Trials	88

Response Profile

Ordered Value	Binary Outcome	Total Frequency
1	Event	62
2	Nonevent	26

Criteria For Assessing Goodness Of Fit

Criterion	DF	Value	Value/DF
Deviance	10	23.9910	2.3991
Scaled Deviance	10	23.9910	2.3991
Pearson Chi-Square	10	21.9206	2.1921
Scaled Pearson X2	10	21.9206	2.1921
Log Likelihood		-60.9970	
Full Log Likelihood		-23.1931	
AIC (smaller is better)		46.3862	
AICC (smaller is better)		46.3862	
BIC (smaller is better)		46.3862	

Algorithm converged.

Analysis Of Maximum Likelihood Parameter Estimates

Parameter	DF	Estimate	Standard Error	Wald 95% Confidence Limits		Wald Chi-Square	Pr > ChiSq
Intercept	0	0.0000	0.0000	0.0000	0.0000	.	.
Scale	0	1.0000	0.0000	1.0000	1.0000		

NOTE: The scale parameter was held fixed.

Lagrange Multiplier Statistics

Parameter	Chi-Square	Pr > ChiSq
Intercept	14.7273	0.0001

Ordinal Quasi Symmetry model, subset: C=3

The GENMOD Procedure

Model Information

Data Set	WORK.GMOCTALLOBS1	
Distribution	Binomial	
Link Function	Logit	
Response Variable (Events)	countYA	Frequency Count
Response Variable (Trials)	nges	

Number of Observations Read	10
Number of Observations Used	10
Number of Events	62
Number of Trials	88

Response Profile

Ordered Value	Binary Outcome	Total Frequency
1	Event	62
2	Nonevent	26

Criteria For Assessing Goodness Of Fit

Criterion	DF	Value	Value/DF
Deviance	9	7.4895	0.8322
Scaled Deviance	9	7.4895	0.8322
Pearson Chi-Square	9	6.9301	0.7700
Scaled Pearson X2	9	6.9301	0.7700
Log Likelihood		-52.7462	
Full Log Likelihood		-14.9423	
AIC (smaller is better)		31.8847	
AICC (smaller is better)		32.3847	
BIC (smaller is better)		32.1872	

Algorithm converged.

Analysis Of Maximum Likelihood Parameter Estimates

Parameter	DF	Estimate	Standard Error	Wald 95% Confidence Limits		Wald Chi-Square	Pr > ChiSq
Intercept	0	0.0000	0.0000	0.0000	0.0000	.	.
score	1	0.4802	0.1311	0.2233	0.7371	13.43	0.0002
Scale	0	1.0000	0.0000	1.0000	1.0000		

NOTE: The scale parameter was held fixed.

Lagrange Multiplier Statistics

Parameter	Chi-Square	Pr > ChiSq
Intercept	0.3306	0.5653

Symmetry, subset: G=1

The GENMOD Procedure

Model Information

Data Set	WORK.GMOCTALLOBS1	
Distribution	Binomial	
Link Function	Logit	
Response Variable (Events)	countYA	Frequency Count
Response Variable (Trials)	nges	

Number of Observations Read	10
Number of Observations Used	10
Number of Events	194
Number of Trials	263

Response Profile

Ordered Value	Binary Outcome	Total Frequency
1	Event	194
2	Nonevent	69

Criteria For Assessing Goodness Of Fit

Criterion	DF	Value	Value/DF
Deviance	10	89.3723	8.9372
Scaled Deviance	10	89.3723	8.9372
Pearson Chi-Square	10	77.7112	7.7711
Scaled Pearson X2	10	77.7112	7.7711
Log Likelihood		-182.2977	
Full Log Likelihood		-58.9634	
AIC (smaller is better)		117.9268	
AICC (smaller is better)		117.9268	
BIC (smaller is better)		117.9268	

Algorithm converged.

Analysis Of Maximum Likelihood Parameter Estimates

Parameter	DF	Estimate	Standard Error	Wald 95% Confidence Limits		Wald Chi-Square	Pr > ChiSq
Intercept	0	0.0000	0.0000	0.0000	0.0000	.	.
Scale	0	1.0000	0.0000	1.0000	1.0000		

NOTE: The scale parameter was held fixed.

Lagrange Multiplier Statistics

Parameter	Chi-Square	Pr > ChiSq
Intercept	59.4106	<.0001

Ordinal Quasi Symmetry model, subset: G=1

The GENMOD Procedure

Model Information

Data Set	WORK.GMOCTALLOBS1	
Distribution	Binomial	
Link Function	Logit	
Response Variable (Events)	countYA	Frequency Count
Response Variable (Trials)	nges	

Number of Observations Read	10
Number of Observations Used	10
Number of Events	194
Number of Trials	263

Response Profile

Ordered Value	Binary Outcome	Total Frequency
1	Event	194
2	Nonevent	69

Criteria For Assessing Goodness Of Fit

Criterion	DF	Value	Value/DF
Deviance	9	17.9395	1.9933
Scaled Deviance	9	17.9395	1.9933
Pearson Chi-Square	9	16.9696	1.8855
Scaled Pearson X2	9	16.9696	1.8855
Log Likelihood		-146.5813	
Full Log Likelihood		-23.2470	
AIC (smaller is better)		48.4940	
AICC (smaller is better)		48.9940	
BIC (smaller is better)		48.7966	

Algorithm converged.

Analysis Of Maximum Likelihood Parameter Estimates

Parameter	DF	Estimate	Standard Error	Wald 95% Confidence Limits		Wald Chi-Square	Pr > ChiSq
Intercept	0	0.0000	0.0000	0.0000	0.0000	.	.
score	1	0.6904	0.0945	0.5052	0.8755	53.42	<.0001
Scale	0	1.0000	0.0000	1.0000	1.0000		

NOTE: The scale parameter was held fixed.

Lagrange Multiplier Statistics

Parameter	Chi-Square	Pr > ChiSq
Intercept	0.1033	0.7479

Symmetry, subset: G=2

The GENMOD Procedure

Model Information

Data Set	WORK.GMOCTALLOBS1	
Distribution	Binomial	
Link Function	Logit	
Response Variable (Events)	countYA	Frequency Count
Response Variable (Trials)	nges	

Number of Observations Read	10
Number of Observations Used	10
Number of Events	255
Number of Trials	375

Response Profile

Ordered Value	Binary Outcome	Total Frequency
1	Event	255
2	Nonevent	120

Criteria For Assessing Goodness Of Fit

Criterion	DF	Value	Value/DF
Deviance	10	65.2361	6.5236
Scaled Deviance	10	65.2361	6.5236
Pearson Chi-Square	10	58.7872	5.8787
Scaled Pearson X2	10	58.7872	5.8787
Log Likelihood		-259.9302	
Full Log Likelihood		-48.2864	
AIC (smaller is better)		96.5728	
AICC (smaller is better)		96.5728	
BIC (smaller is better)		96.5728	

Algorithm converged.

Analysis Of Maximum Likelihood Parameter Estimates

Parameter	DF	Estimate	Standard Error	Wald 95% Confidence Limits		Wald Chi-Square	Pr > ChiSq
Intercept	0	0.0000	0.0000	0.0000	0.0000	.	.
Scale	0	1.0000	0.0000	1.0000	1.0000		

NOTE: The scale parameter was held fixed.

Lagrange Multiplier Statistics

Parameter	Chi-Square	Pr > ChiSq
Intercept	48.6000	<.0001

Ordinal Quasi Symmetry model, subset: G=2

The GENMOD Procedure

Model Information

Data Set	WORK.GMOCTALLOBS1	
Distribution	Binomial	
Link Function	Logit	
Response Variable (Events)	countYA	Frequency Count
Response Variable (Trials)	nges	

Number of Observations Read	10
Number of Observations Used	10
Number of Events	255
Number of Trials	375

Response Profile

Ordered Value	Binary Outcome	Total Frequency
1	Event	255
2	Nonevent	120

Criteria For Assessing Goodness Of Fit

Criterion	DF	Value	Value/DF
Deviance	9	17.1972	1.9108
Scaled Deviance	9	17.1972	1.9108
Pearson Chi-Square	9	13.7402	1.5267
Scaled Pearson X2	9	13.7402	1.5267
Log Likelihood		-235.9107	
Full Log Likelihood		-24.2669	
AIC (smaller is better)		50.5338	
AICC (smaller is better)		51.0338	
BIC (smaller is better)		50.8364	

Algorithm converged.

Analysis Of Maximum Likelihood Parameter Estimates

Parameter	DF	Estimate	Standard Error	Wald 95% Confidence Limits		Wald Chi-Square	Pr > ChiSq
Intercept	0	0.0000	0.0000	0.0000	0.0000	.	.
score	1	0.4869	0.0751	0.3397	0.6341	42.02	<.0001
Scale	0	1.0000	0.0000	1.0000	1.0000		

NOTE: The scale parameter was held fixed.

Lagrange Multiplier Statistics

Parameter	Chi-Square	Pr > ChiSq
Intercept	3.1813	0.0745

Part B: Summary of marginal homogeneity test results

Summary of results of marginal homogeneity tests: ChiSquare tests of restircting Ordered Quasi Symmetry (H1) model to Symmetry (H0) (Agresti, Analysis of Ordinal Categorical Data, 2nd Ed. Section 8.4.7)

subset	Sum Trials	devOQSMod	DFOQSMod	DevSym Mod	Df Sym Mod	dif Deviance	dif DF	pval
R>0	638	24.8502	9	141.418	10	116.568	1	0.00000
R=1	199	13.0227	9	50.576	10	37.553	1	0.00000
R=2	232	15.0944	9	88.996	10	73.902	1	0.00000
R=3	207	17.1025	9	31.995	10	14.892	1	0.00011
C=1	358	22.3601	9	75.267	10	52.907	1	0.00000
C=2	192	8.7303	9	58.743	10	50.013	1	0.00000
C=3	88	7.4895	9	23.991	10	16.502	1	0.00005
G=1	263	17.9395	9	89.372	10	71.433	1	0.00000
G=2	375	17.1972	9	65.236	10	48.039	1	0.00000

Part C: Loglinear model estimation of equation (2) using full data set

The GENMOD Procedure

Model Information

Data Set	WORK.GMOCT
Distribution	Poisson
Link Function	Log
Dependent Variable	COUNT Frequency Count

Number of Observations Read	450
Number of Observations Used	450

Class Level Information

Class	Levels	Values
C	3	1_CZ 2_RU 3_UA
G	2	1_Male 2_Female
R	3	1_BFriend 2_Father 3_Mother
y	5	1_Very bad 2_Bad 3_Neutral 4_Good 5_Very good
z	5	1_Very bad 2_Bad 3_Neutral 4_Good 5_Very good

Criteria For Assessing Goodness Of Fit

Criterion	DF	Value	Value/DF
Deviance	378	385.8993	1.0209
Scaled Deviance	378	385.8993	1.0209
Pearson Chi-Square	378	511.2845	1.3526
Scaled Pearson X2	378	511.2845	1.3526
Log Likelihood		723.5571	
Full Log Likelihood		-607.9588	
AIC (smaller is better)		1359.9175	
AICC (smaller is better)		1387.8008	
BIC (smaller is better)		1655.7834	

Algorithm converged.

Analysis Of Maximum Likelihood Parameter Estimates

Parameter		DF	Estimate	Standard Error	Wald 95% Confidence Limits		Wald Chi-Square	Pr > ChiSq
Intercept		1	-9.3193	2.1111	-13.4570	-5.1815	19.49	<.0001
y	1_Very bad	1	4.4727	1.0207	2.4722	6.4733	19.20	<.0001
y	2_Bad	1	3.2315	0.8924	1.4824	4.9806	13.11	0.0003
y	3_Neutral	1	3.3828	0.6731	2.0636	4.7020	25.26	<.0001
y	4_Good	1	0.8898	0.4959	-0.0821	1.8617	3.22	0.0728
y	5_Very good	0	0.0000	0.0000	0.0000	0.0000	.	.
z	1_Very bad	1	4.9872	1.2363	2.5642	7.4103	16.27	<.0001
z	2_Bad	1	3.7624	1.0812	1.6432	5.8816	12.11	0.0005
z	3_Neutral	1	3.7563	0.8287	2.1320	5.3806	20.54	<.0001
z	4_Good	1	1.6123	0.6032	0.4299	2.7946	7.14	0.0075
z	5_Very good	0	0.0000	0.0000	0.0000	0.0000	.	.
R	1_BFriend	1	1.3970	1.9014	-2.3297	5.1237	0.54	0.4625
R	2_Father	1	0.7398	1.9352	-3.0532	4.5327	0.15	0.7023
R	3_Mother	0	0.0000	0.0000	0.0000	0.0000	.	.
C	1_CZ	1	-4.5137	1.8609	-8.1610	-0.8665	5.88	0.0153
C	2_RU	1	-4.4270	2.0607	-8.4659	-0.3880	4.62	0.0317
C	3_UA	0	0.0000	0.0000	0.0000	0.0000	.	.
G	1_Male	1	3.5031	1.5762	0.4137	6.5924	4.94	0.0263
G	2_Female	0	0.0000	0.0000	0.0000	0.0000	.	.
yz		1	0.3820	0.0877	0.2100	0.5540	18.95	<.0001
yz*C	1_CZ	1	0.1637	0.0766	0.0137	0.3138	4.57	0.0325
yz*C	2_RU	1	0.1549	0.0850	-0.0118	0.3215	3.32	0.0685
yz*C	3_UA	0	0.0000	0.0000	0.0000	0.0000	.	.
yz*G	1_Male	1	-0.0858	0.0651	-0.2133	0.0417	1.74	0.1873
yz*G	2_Female	0	0.0000	0.0000	0.0000	0.0000	.	.

Analysis Of Maximum Likelihood Parameter Estimates

Parameter			DF	Estimate	Standard Error	Wald 95% Confidence Limits		Wald Chi-Square	Pr > ChiSq
yz*R	1_BFriend		1	-0.0710	0.0783	-0.2244	0.0825	0.82	0.3646
yz*R	2_Father		1	-0.0693	0.0790	-0.2241	0.0855	0.77	0.3800
yz*R	3_Mother		0	0.0000	0.0000	0.0000	0.0000	.	.
C*y	1_CZ	1_Very bad	1	2.4168	0.8704	0.7109	4.1228	7.71	0.0055
C*y	1_CZ	2_Bad	1	2.9276	0.7657	1.4268	4.4283	14.62	0.0001
C*y	1_CZ	3_Neutral	1	2.0229	0.5725	0.9008	3.1449	12.49	0.0004
C*y	1_CZ	4_Good	1	1.8003	0.4241	0.9692	2.6315	18.02	<.0001
C*y	1_CZ	5_Very good	0	0.0000	0.0000	0.0000	0.0000	.	.
C*y	2_RU	1_Very bad	1	2.1970	0.9357	0.3631	4.0309	5.51	0.0189
C*y	2_RU	2_Bad	1	2.6887	0.8256	1.0705	4.3068	10.61	0.0011
C*y	2_RU	3_Neutral	1	1.8647	0.6260	0.6377	3.0917	8.87	0.0029
C*y	2_RU	4_Good	1	0.7008	0.4966	-0.2725	1.6740	1.99	0.1582
C*y	2_RU	5_Very good	0	0.0000	0.0000	0.0000	0.0000	.	.
C*y	3_UA	1_Very bad	0	0.0000	0.0000	0.0000	0.0000	.	.
C*y	3_UA	2_Bad	0	0.0000	0.0000	0.0000	0.0000	.	.
C*y	3_UA	3_Neutral	0	0.0000	0.0000	0.0000	0.0000	.	.
C*y	3_UA	4_Good	0	0.0000	0.0000	0.0000	0.0000	.	.
C*y	3_UA	5_Very good	0	0.0000	0.0000	0.0000	0.0000	.	.
G*y	1_Male	1_Very bad	1	-2.2514	0.7212	-3.6650	-0.8378	9.74	0.0018
G*y	1_Male	2_Bad	1	-1.9845	0.6178	-3.1954	-0.7736	10.32	0.0013
G*y	1_Male	3_Neutral	1	-1.7484	0.4764	-2.6822	-0.8147	13.47	0.0002
G*y	1_Male	4_Good	1	-0.8175	0.3448	-1.4932	-0.1417	5.62	0.0177
G*y	1_Male	5_Very good	0	0.0000	0.0000	0.0000	0.0000	.	.
G*y	2_Female	1_Very bad	0	0.0000	0.0000	0.0000	0.0000	.	.
G*y	2_Female	2_Bad	0	0.0000	0.0000	0.0000	0.0000	.	.
G*y	2_Female	3_Neutral	0	0.0000	0.0000	0.0000	0.0000	.	.
G*y	2_Female	4_Good	0	0.0000	0.0000	0.0000	0.0000	.	.
G*y	2_Female	5_Very good	0	0.0000	0.0000	0.0000	0.0000	.	.
R*y	1_BFriend	1_Very bad	1	-0.9583	0.8920	-2.7065	0.7900	1.15	0.2827
R*y	1_BFriend	2_Bad	1	-0.7518	0.7635	-2.2483	0.7447	0.97	0.3248
R*y	1_BFriend	3_Neutral	1	-0.5156	0.5871	-1.6664	0.6351	0.77	0.3798
R*y	1_BFriend	4_Good	1	-0.2622	0.4183	-1.0821	0.5576	0.39	0.5308
R*y	1_BFriend	5_Very good	0	0.0000	0.0000	0.0000	0.0000	.	.
R*y	2_Father	1_Very bad	1	-1.2564	0.8752	-2.9716	0.4589	2.06	0.1511
R*y	2_Father	2_Bad	1	-0.9952	0.7490	-2.4632	0.4727	1.77	0.1839
R*y	2_Father	3_Neutral	1	-0.7002	0.5756	-1.8284	0.4280	1.48	0.2238
R*y	2_Father	4_Good	1	-0.3851	0.4109	-1.1905	0.4203	0.88	0.3487
R*y	2_Father	5_Very good	0	0.0000	0.0000	0.0000	0.0000	.	.
R*y	3_Mother	1_Very bad	0	0.0000	0.0000	0.0000	0.0000	.	.
R*y	3_Mother	2_Bad	0	0.0000	0.0000	0.0000	0.0000	.	.
R*y	3_Mother	3_Neutral	0	0.0000	0.0000	0.0000	0.0000	.	.
R*y	3_Mother	4_Good	0	0.0000	0.0000	0.0000	0.0000	.	.
R*y	3_Mother	5_Very good	0	0.0000	0.0000	0.0000	0.0000	.	.
C*z	1_CZ	1_Very bad	1	3.3810	1.1256	1.1748	5.5871	9.02	0.0027
C*z	1_CZ	2_Bad	1	4.2614	0.9854	2.3301	6.1927	18.70	<.0001
C*z	1_CZ	3_Neutral	1	3.1607	0.7552	1.6806	4.6409	17.52	<.0001
C*z	1_CZ	4_Good	1	2.2647	0.5603	1.1665	3.3629	16.34	<.0001
C*z	1_CZ	5_Very good	0	0.0000	0.0000	0.0000	0.0000	.	.
C*z	2_RU	1_Very bad	1	3.5027	1.2539	1.0451	5.9603	7.80	0.0052
C*z	2_RU	2_Bad	1	3.7685	1.0959	1.6206	5.9165	11.82	0.0006
C*z	2_RU	3_Neutral	1	2.5708	0.8457	0.9132	4.2284	9.24	0.0024
C*z	2_RU	4_Good	1	1.1089	0.6495	-0.1641	2.3819	2.91	0.0878
C*z	2_RU	5_Very good	0	0.0000	0.0000	0.0000	0.0000	.	.
C*z	3_UA	1_Very bad	0	0.0000	0.0000	0.0000	0.0000	.	.
C*z	3_UA	2_Bad	0	0.0000	0.0000	0.0000	0.0000	.	.
C*z	3_UA	3_Neutral	0	0.0000	0.0000	0.0000	0.0000	.	.
C*z	3_UA	4_Good	0	0.0000	0.0000	0.0000	0.0000	.	.
C*z	3_UA	5_Very good	0	0.0000	0.0000	0.0000	0.0000	.	.
G*z	1_Male	1_Very bad	1	-1.5925	0.9448	-3.4443	0.2593	2.84	0.0919
G*z	1_Male	2_Bad	1	-1.3847	0.8180	-2.9879	0.2185	2.87	0.0905
G*z	1_Male	3_Neutral	1	-0.9668	0.6452	-2.2315	0.2978	2.25	0.1340
G*z	1_Male	4_Good	1	-0.7319	0.4847	-1.6819	0.2181	2.28	0.1311
G*z	1_Male	5_Very good	0	0.0000	0.0000	0.0000	0.0000	.	.
G*z	2_Female	1_Very bad	0	0.0000	0.0000	0.0000	0.0000	.	.
G*z	2_Female	2_Bad	0	0.0000	0.0000	0.0000	0.0000	.	.
G*z	2_Female	3_Neutral	0	0.0000	0.0000	0.0000	0.0000	.	.
G*z	2_Female	4_Good	0	0.0000	0.0000	0.0000	0.0000	.	.
G*z	2_Female	5_Very good	0	0.0000	0.0000	0.0000	0.0000	.	.
R*z	1_BFriend	1_Very bad	1	-0.1147	1.1040	-2.2785	2.0492	0.01	0.9173
R*z	1_BFriend	2_Bad	1	-0.6702	0.9572	-2.5462	1.2058	0.49	0.4838
R*z	1_BFriend	3_Neutral	1	-0.4292	0.7535	-1.9061	1.0477	0.32	0.5690
R*z	1_BFriend	4_Good	1	-0.2267	0.5523	-1.3093	0.8558	0.17	0.6814
R*z	1_BFriend	5_Very good	0	0.0000	0.0000	0.0000	0.0000	.	.
R*z	2_Father	1_Very bad	1	0.9325	1.1612	-1.3434	3.2084	0.64	0.4219
R*z	2_Father	2_Bad	1	0.3026	1.0127	-1.6822	2.2875	0.09	0.7651

Analysis Of Maximum Likelihood Parameter Estimates

Parameter				DF	Estimate	Standard Error	Wald 95% Confidence Limits		Wald Chi-Square	Pr > ChiSq
R*z	2_Father	3_Neutral		1	0.3564	0.8103	-1.2318	1.9447	0.19	0.6600
R*z	2_Father	4_Good		1	0.4579	0.6147	-0.7470	1.6628	0.55	0.4564
R*z	2_Father	5_Very good		0	0.0000	0.0000	0.0000	0.0000	.	.
R*z	3_Mother	1_Very bad		0	0.0000	0.0000	0.0000	0.0000	.	.
R*z	3_Mother	2_Bad		0	0.0000	0.0000	0.0000	0.0000	.	.
R*z	3_Mother	3_Neutral		0	0.0000	0.0000	0.0000	0.0000	.	.
R*z	3_Mother	4_Good		0	0.0000	0.0000	0.0000	0.0000	.	.
R*z	3_Mother	5_Very good		0	0.0000	0.0000	0.0000	0.0000	.	.
C*G	1_CZ	1_Male		1	-0.8823	0.3323	-1.5336	-0.2309	7.05	0.0079
C*G	1_CZ	2_Female		0	0.0000	0.0000	0.0000	0.0000	.	.
C*G	2_RU	1_Male		1	-0.6082	0.3567	-1.3072	0.0908	2.91	0.0881
C*G	2_RU	2_Female		0	0.0000	0.0000	0.0000	0.0000	.	.
C*G	3_UA	1_Male		0	0.0000	0.0000	0.0000	0.0000	.	.
C*G	3_UA	2_Female		0	0.0000	0.0000	0.0000	0.0000	.	.
C*R	1_CZ	1_BFriend		1	0.0997	0.3473	-0.5810	0.7803	0.08	0.7741
C*R	1_CZ	2_Father		1	0.0850	0.3484	-0.5980	0.7679	0.06	0.8073
C*R	1_CZ	3_Mother		0	0.0000	0.0000	0.0000	0.0000	.	.
C*R	2_RU	1_BFriend		1	-0.0042	0.3680	-0.7254	0.7170	0.00	0.9909
C*R	2_RU	2_Father		1	-0.0395	0.3687	-0.7622	0.6833	0.01	0.9147
C*R	2_RU	3_Mother		0	0.0000	0.0000	0.0000	0.0000	.	.
C*R	3_UA	1_BFriend		0	0.0000	0.0000	0.0000	0.0000	.	.
C*R	3_UA	2_Father		0	0.0000	0.0000	0.0000	0.0000	.	.
C*R	3_UA	3_Mother		0	0.0000	0.0000	0.0000	0.0000	.	.
G*R	1_Male	1_BFriend		1	0.0975	0.4143	-0.7144	0.9094	0.06	0.8139
G*R	1_Male	2_Father		1	0.1562	0.4149	-0.6571	0.9695	0.14	0.7066
G*R	1_Male	3_Mother		0	0.0000	0.0000	0.0000	0.0000	.	.
G*R	2_Female	1_BFriend		0	0.0000	0.0000	0.0000	0.0000	.	.
G*R	2_Female	2_Father		0	0.0000	0.0000	0.0000	0.0000	.	.
G*R	2_Female	3_Mother		0	0.0000	0.0000	0.0000	0.0000	.	.
C*G*R	1_CZ	1_Male	1_BFriend	1	-0.0792	0.4602	-0.9812	0.8227	0.03	0.8633
C*G*R	1_CZ	1_Male	2_Father	1	-0.1438	0.4597	-1.0448	0.7573	0.10	0.7545
C*G*R	1_CZ	1_Male	3_Mother	0	0.0000	0.0000	0.0000	0.0000	.	.
C*G*R	1_CZ	2_Female	1_BFriend	0	0.0000	0.0000	0.0000	0.0000	.	.
C*G*R	1_CZ	2_Female	2_Father	0	0.0000	0.0000	0.0000	0.0000	.	.
C*G*R	1_CZ	2_Female	3_Mother	0	0.0000	0.0000	0.0000	0.0000	.	.
C*G*R	2_RU	1_Male	1_BFriend	1	-0.0676	0.4954	-1.0386	0.9034	0.02	0.8914
C*G*R	2_RU	1_Male	2_Father	1	-0.1236	0.4943	-1.0925	0.8452	0.06	0.8025
C*G*R	2_RU	1_Male	3_Mother	0	0.0000	0.0000	0.0000	0.0000	.	.
C*G*R	2_RU	2_Female	1_BFriend	0	0.0000	0.0000	0.0000	0.0000	.	.
C*G*R	2_RU	2_Female	2_Father	0	0.0000	0.0000	0.0000	0.0000	.	.
C*G*R	2_RU	2_Female	3_Mother	0	0.0000	0.0000	0.0000	0.0000	.	.
C*G*R	3_UA	1_Male	1_BFriend	0	0.0000	0.0000	0.0000	0.0000	.	.
C*G*R	3_UA	1_Male	2_Father	0	0.0000	0.0000	0.0000	0.0000	.	.
C*G*R	3_UA	1_Male	3_Mother	0	0.0000	0.0000	0.0000	0.0000	.	.
C*G*R	3_UA	2_Female	1_BFriend	0	0.0000	0.0000	0.0000	0.0000	.	.
C*G*R	3_UA	2_Female	2_Father	0	0.0000	0.0000	0.0000	0.0000	.	.
C*G*R	3_UA	2_Female	3_Mother	0	0.0000	0.0000	0.0000	0.0000	.	.
Scale				0	1.0000	0.0000	1.0000	1.0000		

NOTE: The scale parameter was held fixed.

Contrast Estimate Results

Label	Mean Estimate	Mean Confidence Limits		L'Beta Estimate	Standard Error	Alpha	L'Beta Confidence Limits		Chi-Square
(male-female)*acay	0.9178	0.8079	1.0426	-0.0858	0.0651	0.05	-0.2133	0.0417	1.74
(CZ-RU)*yz	1.0089	0.8662	1.1752	0.0089	0.0778	0.05	-0.1437	0.1614	0.01
(CZ-UA)*yz	1.1779	1.0138	1.3686	0.1637	0.0766	0.05	0.0137	0.3138	4.57
(RU-UA)*yz	0.8565	0.7251	1.0118	-0.1549	0.0850	0.05	-0.3215	0.0118	3.32
(BF-Fa)*yz	0.9984	0.8627	1.1553	-0.0016	0.0745	0.05	-0.1477	0.1444	0.00
(BF-Mo)*yz	0.9315	0.7990	1.0860	-0.0710	0.0783	0.05	-0.2244	0.0825	0.82
(Fa-Mo)*yz	1.0718	0.9181	1.2512	0.0693	0.0790	0.05	-0.0855	0.2241	0.77

Label	Pr > ChiSq
(male-female)*acay	0.1873
(CZ-RU)*yz	0.9093
(CZ-UA)*yz	0.0325
(RU-UA)*yz	0.0685
(BF-Fa)*yz	0.9824
(BF-Mo)*yz	0.3646
(Fa-Mo)*yz	0.3800

Part D: Final loglinear model estimation of equation (2) as excerpted in Table 4

The GENMOD Procedure

Model Information

Data Set	WORK.GMOCT	
Distribution	Poisson	
Link Function	Log	
Dependent Variable	COUNT	Frequency Count

Number of Observations Read	450
Number of Observations Used	450

Class Level Information

Class	Levels	Values
C	3	1_CZ 2_RU 3_UA
G	2	1_Male 2_Female
R	3	1_BFriend 2_Father 3_Mother
y	5	1_Very bad 2_Bad 3_Neutral 4_Good 5_Very good
z	5	1_Very bad 2_Bad 3_Neutral 4_Good 5_Very good

Criteria For Assessing Goodness Of Fit

Criterion	DF	Value	Value/DF
Deviance	378	351.5310	0.9300
Scaled Deviance	378	351.5310	0.9300
Pearson Chi-Square	378	354.7109	0.9384
Scaled Pearson X2	378	354.7109	0.9384
Log Likelihood		746.3549	
Full Log Likelihood		-584.4678	
AIC (smaller is better)		1312.9355	
AICC (smaller is better)		1340.8188	
BIC (smaller is better)		1608.8013	

Algorithm converged.

Analysis Of Maximum Likelihood Parameter Estimates

Parameter		DF	Estimate	Standard Error	Wald 95% Confidence Limits		Wald Chi-Square	Pr > ChiSq
Intercept		1	-12.2867	2.3163	-16.8266	-7.7467	28.14	<.0001
y	1_Very bad	1	6.0203	1.1117	3.8414	8.1992	29.33	<.0001
y	2_Bad	1	4.5938	0.9740	2.6847	6.5028	22.24	<.0001
y	3_Neutral	1	4.4323	0.7369	2.9880	5.8765	36.18	<.0001
y	4_Good	1	1.5432	0.5263	0.5118	2.5747	8.60	0.0034
y	5_Very good	0	0.0000	0.0000	0.0000	0.0000	.	.
z	1_Very bad	1	6.3081	1.3353	3.6910	8.9252	22.32	<.0001
z	2_Bad	1	4.7711	1.1740	2.4701	7.0720	16.52	<.0001
z	3_Neutral	1	4.6407	0.8994	2.8779	6.4036	26.62	<.0001
z	4_Good	1	2.1189	0.6427	0.8593	3.3785	10.87	0.0010
z	5_Very good	0	0.0000	0.0000	0.0000	0.0000	.	.
R	1_BFriend	1	3.8663	2.0379	-0.1279	7.8605	3.60	0.0578
R	2_Father	1	2.1752	2.1215	-1.9828	6.3333	1.05	0.3052
R	3_Mother	0	0.0000	0.0000	0.0000	0.0000	.	.
C	1_CZ	1	-4.2637	1.9207	-8.0281	-0.4992	4.93	0.0264
C	2_RU	1	-5.7710	2.2175	-10.1172	-1.4247	6.77	0.0093
C	3_UA	0	0.0000	0.0000	0.0000	0.0000	.	.
G	1_Male	1	4.8791	1.6628	1.6201	8.1381	8.61	0.0033
G	2_Female	0	0.0000	0.0000	0.0000	0.0000	.	.
yz		1	0.4980	0.0956	0.3106	0.6853	27.14	<.0001
yz*C	1_CZ	1	0.1544	0.0786	0.0004	0.3085	3.86	0.0494
yz*C	2_RU	1	0.2057	0.0905	0.0283	0.3832	5.16	0.0231
yz*C	3_UA	0	0.0000	0.0000	0.0000	0.0000	.	.
yz*G	1_Male	1	-0.1350	0.0682	-0.2687	-0.0014	3.92	0.0477
yz*G	2_Female	0	0.0000	0.0000	0.0000	0.0000	.	.
yz*R	1_BFriend	1	-0.1664	0.0835	-0.3301	-0.0027	3.97	0.0463
yz*R	2_Father	1	-0.1323	0.0858	-0.3005	0.0358	2.38	0.1229

Analysis Of Maximum Likelihood Parameter Estimates

Parameter			DF	Estimate	Standard Error	Wald 95% Confidence Limits		Wald Chi-Square	Pr > ChiSq
yz*R	3_Mother		0	0.0000	0.0000	0.0000	0.0000	.	.
C*y	1_CZ	1_Very bad	1	2.2179	0.8919	0.4698	3.9660	6.18	0.0129
C*y	1_CZ	2_Bad	1	2.7337	0.7868	1.1916	4.2758	12.07	0.0005
C*y	1_CZ	3_Neutral	1	1.8691	0.5896	0.7135	3.0247	10.05	0.0015
C*y	1_CZ	4_Good	1	1.6987	0.4317	0.8527	2.5447	15.49	<.0001
C*y	1_CZ	5_Very good	0	0.0000	0.0000	0.0000	0.0000	.	.
C*y	2_RU	1_Very bad	1	2.5324	0.9861	0.5996	4.4653	6.59	0.0102
C*y	2_RU	2_Bad	1	2.9701	0.8724	1.2602	4.6800	11.59	0.0007
C*y	2_RU	3_Neutral	1	2.0795	0.6628	0.7804	3.3786	9.84	0.0017
C*y	2_RU	4_Good	1	0.8110	0.5116	-0.1917	1.8137	2.51	0.1129
C*y	2_RU	5_Very good	0	0.0000	0.0000	0.0000	0.0000	.	.
C*y	3_UA	1_Very bad	0	0.0000	0.0000	0.0000	0.0000	.	.
C*y	3_UA	2_Bad	0	0.0000	0.0000	0.0000	0.0000	.	.
C*y	3_UA	3_Neutral	0	0.0000	0.0000	0.0000	0.0000	.	.
C*y	3_UA	4_Good	0	0.0000	0.0000	0.0000	0.0000	.	.
C*y	3_UA	5_Very good	0	0.0000	0.0000	0.0000	0.0000	.	.
G*y	1_Male	1_Very bad	1	-2.8752	0.7541	-4.3532	-1.3973	14.54	0.0001
G*y	1_Male	2_Bad	1	-2.5463	0.6485	-3.8174	-1.2752	15.42	<.0001
G*y	1_Male	3_Neutral	1	-2.1882	0.5005	-3.1691	-1.2073	19.12	<.0001
G*y	1_Male	4_Good	1	-1.1018	0.3572	-1.8018	-0.4018	9.52	0.0020
G*y	1_Male	5_Very good	0	0.0000	0.0000	0.0000	0.0000	.	.
G*y	2_Female	1_Very bad	0	0.0000	0.0000	0.0000	0.0000	.	.
G*y	2_Female	2_Bad	0	0.0000	0.0000	0.0000	0.0000	.	.
G*y	2_Female	3_Neutral	0	0.0000	0.0000	0.0000	0.0000	.	.
G*y	2_Female	4_Good	0	0.0000	0.0000	0.0000	0.0000	.	.
G*y	2_Female	5_Very good	0	0.0000	0.0000	0.0000	0.0000	.	.
R*y	1_BFriend	1_Very bad	1	-2.1282	0.9544	-3.9989	-0.2576	4.97	0.0258
R*y	1_BFriend	2_Bad	1	-1.7626	0.8192	-3.3682	-0.1570	4.63	0.0314
R*y	1_BFriend	3_Neutral	1	-1.2865	0.6289	-2.5192	-0.0538	4.18	0.0408
R*y	1_BFriend	4_Good	1	-0.7232	0.4383	-1.5823	0.1360	2.72	0.0990
R*y	1_BFriend	5_Very good	0	0.0000	0.0000	0.0000	0.0000	.	.
R*y	2_Father	1_Very bad	1	-2.1449	0.9503	-4.0076	-0.2823	5.09	0.0240
R*y	2_Father	2_Bad	1	-1.7916	0.8167	-3.3922	-0.1909	4.81	0.0283
R*y	2_Father	3_Neutral	1	-1.2918	0.6265	-2.5197	-0.0638	4.25	0.0392
R*y	2_Father	4_Good	1	-0.7516	0.4348	-1.6038	0.1005	2.99	0.0838
R*y	2_Father	5_Very good	0	0.0000	0.0000	0.0000	0.0000	.	.
R*y	3_Mother	1_Very bad	0	0.0000	0.0000	0.0000	0.0000	.	.
R*y	3_Mother	2_Bad	0	0.0000	0.0000	0.0000	0.0000	.	.
R*y	3_Mother	3_Neutral	0	0.0000	0.0000	0.0000	0.0000	.	.
R*y	3_Mother	4_Good	0	0.0000	0.0000	0.0000	0.0000	.	.
R*y	3_Mother	5_Very good	0	0.0000	0.0000	0.0000	0.0000	.	.
C*z	1_CZ	1_Very bad	1	3.3877	1.1547	1.1244	5.6509	8.61	0.0033
C*z	1_CZ	2_Bad	1	4.4118	1.0161	2.4203	6.4033	18.85	<.0001
C*z	1_CZ	3_Neutral	1	3.1856	0.7773	1.6621	4.7092	16.80	<.0001
C*z	1_CZ	4_Good	1	2.3111	0.5749	1.1842	3.4379	16.16	<.0001
C*z	1_CZ	5_Very good	0	0.0000	0.0000	0.0000	0.0000	.	.
C*z	2_RU	1_Very bad	1	4.5141	1.3486	1.8708	7.1574	11.20	0.0008
C*z	2_RU	2_Bad	1	4.7923	1.1850	2.4698	7.1149	16.36	<.0001
C*z	2_RU	3_Neutral	1	3.2934	0.9178	1.4946	5.0923	12.88	0.0003
C*z	2_RU	4_Good	1	1.6239	0.6959	0.2600	2.9879	5.45	0.0196
C*z	2_RU	5_Very good	0	0.0000	0.0000	0.0000	0.0000	.	.
C*z	3_UA	1_Very bad	0	0.0000	0.0000	0.0000	0.0000	.	.
C*z	3_UA	2_Bad	0	0.0000	0.0000	0.0000	0.0000	.	.
C*z	3_UA	3_Neutral	0	0.0000	0.0000	0.0000	0.0000	.	.
C*z	3_UA	4_Good	0	0.0000	0.0000	0.0000	0.0000	.	.
C*z	3_UA	5_Very good	0	0.0000	0.0000	0.0000	0.0000	.	.
G*z	1_Male	1_Very bad	1	-2.2102	0.9930	-4.1564	-0.2639	4.95	0.0260
G*z	1_Male	2_Bad	1	-1.9051	0.8619	-3.5944	-0.2159	4.89	0.0271
G*z	1_Male	3_Neutral	1	-1.3850	0.6810	-2.7197	-0.0504	4.14	0.0420
G*z	1_Male	4_Good	1	-0.9959	0.5087	-1.9929	0.0011	3.83	0.0503
G*z	1_Male	5_Very good	0	0.0000	0.0000	0.0000	0.0000	.	.
G*z	2_Female	1_Very bad	0	0.0000	0.0000	0.0000	0.0000	.	.
G*z	2_Female	2_Bad	0	0.0000	0.0000	0.0000	0.0000	.	.
G*z	2_Female	3_Neutral	0	0.0000	0.0000	0.0000	0.0000	.	.
G*z	2_Female	4_Good	0	0.0000	0.0000	0.0000	0.0000	.	.
G*z	2_Female	5_Very good	0	0.0000	0.0000	0.0000	0.0000	.	.
R*z	1_BFriend	1_Very bad	1	-1.3032	1.1683	-3.5930	0.9867	1.24	0.2647
R*z	1_BFriend	2_Bad	1	-1.7015	1.0164	-3.6935	0.2906	2.80	0.0941
R*z	1_BFriend	3_Neutral	1	-1.2341	0.7990	-2.8001	0.3319	2.39	0.1225
R*z	1_BFriend	4_Good	1	-0.7000	0.5755	-1.8280	0.4280	1.48	0.2239
R*z	1_BFriend	5_Very good	0	0.0000	0.0000	0.0000	0.0000	.	.
R*z	2_Father	1_Very bad	1	0.5227	1.2664	-1.9595	3.0049	0.17	0.6798
R*z	2_Father	2_Bad	1	-0.0224	1.1113	-2.2005	2.1556	0.00	0.9839
R*z	2_Father	3_Neutral	1	0.1510	0.8933	-1.5999	1.9020	0.03	0.8657
R*z	2_Father	4_Good	1	0.4499	0.6744	-0.8719	1.7717	0.45	0.5047
R*z	2_Father	5_Very good	0	0.0000	0.0000	0.0000	0.0000	.	.
R*z	3_Mother	1_Very bad	0	0.0000	0.0000	0.0000	0.0000	.	.
R*z	3_Mother	2_Bad	0	0.0000	0.0000	0.0000	0.0000	.	.

R*z	3_Mother	3_Neutral		0	0.0000	0.0000	0.0000	0.0000	.	.

Analysis Of Maximum Likelihood Parameter Estimates

Parameter				DF	Estimate	Standard Error	Wald 95% Confidence Limits		Wald Chi-Square	Pr > ChiSq
R*z	3_Mother	4_Good		0	0.0000	0.0000	0.0000	0.0000	.	.
R*z	3_Mother	5_Very good		0	0.0000	0.0000	0.0000	0.0000	.	.
C*G	1_CZ	1_Male		1	-0.9716	0.3416	-1.6411	-0.3021	8.09	0.0044
C*G	1_CZ	2_Female		0	0.0000	0.0000	0.0000	0.0000	.	.
C*G	2_RU	1_Male		1	-0.6730	0.3662	-1.3908	0.0448	3.38	0.0661
C*G	2_RU	2_Female		0	0.0000	0.0000	0.0000	0.0000	.	.
C*G	3_UA	1_Male		0	0.0000	0.0000	0.0000	0.0000	.	.
C*G	3_UA	2_Female		0	0.0000	0.0000	0.0000	0.0000	.	.
C*R	1_CZ	1_BFriend		1	0.0249	0.3556	-0.6721	0.7218	0.00	0.9442
C*R	1_CZ	2_Father		1	-0.0199	0.3570	-0.7197	0.6799	0.00	0.9555
C*R	1_CZ	3_Mother		0	0.0000	0.0000	0.0000	0.0000	.	.
C*R	2_RU	1_BFriend		1	-0.0579	0.3766	-0.7961	0.6803	0.02	0.8778
C*R	2_RU	2_Father		1	-0.1217	0.3776	-0.8618	0.6183	0.10	0.7471
C*R	2_RU	3_Mother		0	0.0000	0.0000	0.0000	0.0000	.	.
C*R	3_UA	1_BFriend		0	0.0000	0.0000	0.0000	0.0000	.	.
C*R	3_UA	2_Father		0	0.0000	0.0000	0.0000	0.0000	.	.
C*R	3_UA	3_Mother		0	0.0000	0.0000	0.0000	0.0000	.	.
G*R	1_Male	1_BFriend		1	-0.0421	0.4218	-0.8689	0.7847	0.01	0.9204
G*R	1_Male	2_Father		1	0.0329	0.4234	-0.7970	0.8627	0.01	0.9381
G*R	1_Male	3_Mother		0	0.0000	0.0000	0.0000	0.0000	.	.
G*R	2_Female	1_BFriend		0	0.0000	0.0000	0.0000	0.0000	.	.
G*R	2_Female	2_Father		0	0.0000	0.0000	0.0000	0.0000	.	.
G*R	2_Female	3_Mother		0	0.0000	0.0000	0.0000	0.0000	.	.
C*G*R	1_CZ	1_Male	1_BFriend	1	0.0301	0.4667	-0.8847	0.9448	0.00	0.9487
C*G*R	1_CZ	1_Male	2_Father	1	-0.0467	0.4669	-0.9619	0.8685	0.01	0.9203
C*G*R	1_CZ	1_Male	3_Mother	0	0.0000	0.0000	0.0000	0.0000	.	.
C*G*R	1_CZ	2_Female	1_BFriend	0	0.0000	0.0000	0.0000	0.0000	.	.
C*G*R	1_CZ	2_Female	2_Father	0	0.0000	0.0000	0.0000	0.0000	.	.
C*G*R	1_CZ	2_Female	3_Mother	0	0.0000	0.0000	0.0000	0.0000	.	.
C*G*R	2_RU	1_Male	1_BFriend	1	0.0169	0.5019	-0.9668	1.0006	0.00	0.9731
C*G*R	2_RU	1_Male	2_Father	1	-0.0796	0.5020	-1.0636	0.9043	0.03	0.8740
C*G*R	2_RU	1_Male	3_Mother	0	0.0000	0.0000	0.0000	0.0000	.	.
C*G*R	2_RU	2_Female	1_BFriend	0	0.0000	0.0000	0.0000	0.0000	.	.
C*G*R	2_RU	2_Female	2_Father	0	0.0000	0.0000	0.0000	0.0000	.	.
C*G*R	2_RU	2_Female	3_Mother	0	0.0000	0.0000	0.0000	0.0000	.	.
C*G*R	3_UA	1_Male	1_BFriend	0	0.0000	0.0000	0.0000	0.0000	.	.
C*G*R	3_UA	1_Male	2_Father	0	0.0000	0.0000	0.0000	0.0000	.	.
C*G*R	3_UA	1_Male	3_Mother	0	0.0000	0.0000	0.0000	0.0000	.	.
C*G*R	3_UA	2_Female	1_BFriend	0	0.0000	0.0000	0.0000	0.0000	.	.
C*G*R	3_UA	2_Female	2_Father	0	0.0000	0.0000	0.0000	0.0000	.	.
C*G*R	3_UA	2_Female	3_Mother	0	0.0000	0.0000	0.0000	0.0000	.	.
Scale				0	1.0000	0.0000	1.0000	1.0000		

NOTE: The scale parameter was held fixed.

Contrast Estimate Results

Label	Mean Estimate	Mean Confidence Limits		L'Beta Estimate	Standard Error	Alpha	L'Beta Confidence Limits		Chi-Square	Pr>ChiSq
(male-female)*acay	0.874	0.764	0.999	-0.135	0.068	0.05	-0.269	-0.001	3.92	0.048
(CZ-RU)*yz	0.950	0.807	1.118	-0.051	0.083	0.05	-0.214	0.112	0.38	0.537
(CZ-UA)*yz	1.167	1.000	1.361	0.154	0.079	0.05	0.000	0.309	3.86	0.049
(RU-UA)*yz	0.814	0.682	0.972	-0.206	0.091	0.05	-0.383	-0.028	5.16	0.023
(BF-Fa)*yz	0.967	0.833	1.122	-0.034	0.076	0.05	-0.183	0.115	0.20	0.654
(BF-Mo)*yz	0.847	0.719	0.997	-0.166	0.084	0.05	-0.330	-0.003	3.97	0.046
(Fa-Mo)*yz	1.142	0.965	1.351	0.132	0.086	0.05	-0.036	0.301	2.38	0.123
